# Linking Metastatic Behavior and Metabolic Heterogeneity of Circulating Tumor Cells at Single‐Cell Level Using an Integrative Microfluidic System

**DOI:** 10.1002/advs.202413978

**Published:** 2025-02-17

**Authors:** Ying Hou, Jiaxu Lin, Hongren Yao, Zengnan Wu, Yongning Lin, Jin‐Ming Lin

**Affiliations:** ^1^ Department of Chemistry Beijing Key Laboratory of Microanalytical Methods and Instrumentation Key Laboratory of Bioorganic Phosphorus Chemistry & Chemical Biology (Ministry of Education) Tsinghua University Beijing 100084 China

**Keywords:** circulating tumor cells, integrative analysis, metastasis, microfluidics, single‐cell

## Abstract

Circulating tumor cells (CTCs) are pivotal biomarkers in tumor metastasis, however, the underlying molecular mechanism of CTCs behavioral heterogeneity during metastasis remains unexplored. Here, an integrative workflow is developed to link behavior characteristics to metabolic profiling within individual CTCs, which simulates the metastatic process on a microfluidic system and combined with single‐cell mass spectrometry (MS) detection. Spheroid‐derived HCT116 cells are tracked and extracted via a temporary vascular system, revealing various arrest patterns under biomimetic vascular shear flow. Downstream MS analysis characterizes 17 cellular metabolites and associates metabolic profiles with de‐adhesion behaviors of the same CTCs, identifying a potential high‐metastatic subpopulation with enhanced arrest ability and evaluating critical metabolites involved in metastasis pathways. Additionally, the metastasis‐inhibiting effect of anti‐tumor drug 5‐fluorouracil by reducing high‐metastatic cells in spheroids is elucidated. This approach offers a valuable opportunity to dissect the interplay of the metastatic behavior and metabolic profiles of CTCs and foster insights into the molecular mechanisms underlying behavioral phenotypes in the tumor metastasis process.

## Graphical abstract

An integrative workflow is developed to investigate the underlying associations between the metastasis behavior and metabolic characteristics of circulating tumor cells, through tracking the metastatic process on microfluidic chips and combining with mass spectrometry for single‐cell profiling. The bioinformatics analysis uncovers the related metabolic pathways and a potential high metastatic subpopulation.

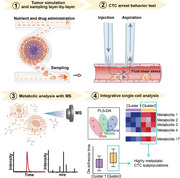

## 1 Introduction

Metastasis remains the primary cause of death in cancer patients.^[^ ^1^ ^]^ The process of blood‐borne metastasis initiates when circulating tumor cells (CTCs) dissociate from the primary tumor, intravasate into the bloodstream, arrest within blood vessels as single cells or clusters, and eventually extravasate to establish metastatic sites.^[^ ^2^ ^]^ CTCs, as the central mediators in metastasis, hold significant potential for advancing early diagnosis, therapeutic strategies, and prognostic assessments in oncology.^[^ ^3^, ^4^ ^]^ However, the inherent heterogeneity, fragility, and rarity of CTCs have impeded in‐depth investigations into the physiological mechanisms driving metastasis.^[^ ^5^ ^]^ Despite significant progress in single‐cell omics technologies, such as single‐cell transcriptomics and proteomics, which facilitate the analysis of preclinical models and clinical cancer specimens,^[^ ^6^ ^]^ directly tracing the intricate behavioral and molecular mechanisms of CTCs remains challenging. Therefore, it is imperative to develop analytical methodologies that enable concurrent examination of the molecular foundations and metastatic behaviors of CTCs at the single‐cell level.

Research on CTCs in tumor metastasis has undergone remarkable advancements in recent decades, with methodologies broadly categorized into two groups. One focuses on rapid CTC capture‐detection technologies for liquid biopsy and clinical diagnostics. Employing antigen‐dependent or antigen‐independent CTC capture techniques, these methods enable the identification and analysis of CTCs using single‐cell molecular detection technologies.^[^ ^7^, ^8^ ^]^ The primary advantage lies in their physiological relevance, showing promise for prognosis management and identification of therapeutic targets.^[^ ^9^ ^]^ However, limited information is offered due to their poor compatibility with various molecular assays. Moreover, the challenge of long‐term survival of CTCs in vitro complicates the acquisition of data on their behavioral variability.^[^ ^5^ ^]^ Another category of techniques emphasizes the mechanistic study of the CTC metastasis process, aiming to elucidate the link between cellular behavioral phenotypes and their underlying biological foundations.^[^ ^10^ ^]^ Typically, in vitro metastasis models are employed, integrating cell morphology imaging, cell capture, and single‐cell analysis to investigate the metastatic process.^[^ ^11^, ^12^ ^]^ However, most current models are relatively simple (e.g., scratch assays and transwell assays), raising questions regarding their applicability to clinical samples. Nonetheless, the combination of single‐cell functional and molecular analysis allows for the exploration of the driving forces behind specific cellular patterns^[^ ^13^, ^14^ ^]^ and is increasingly employed in diverse tissue and disease investigations.^[^ ^15^ ^]^

It is noteworthy that though ≈10^6^ cells are shed from the primary tumor every day, the successful metastasis rate remains below 0.01%.^[^ ^16^ ^]^ In fact, clinical evidence indicates that the quantity of CTCs in patient blood is not directly correlated with metastatic propensity.^[^ ^17^ ^]^ This result arises from the majority of CTCs succumbing to fluid shear stress within the vasculature, while only a minority can adhere to the vascular wall and accomplish metastasis. Although the stalling behavior of CTCs is considered a prerequisite for tumor metastasis, the underlying mechanisms remain incompletely understood. Moreover, the heterogeneity of CTCs suggests a potential association of this behavior with specific cellular subpopulations.^[^ ^18^ ^]^ This underscores the importance of investigating CTC subpopulations with metastatic potential under physiological conditions and elucidating their molecular characteristics for the development of anti‐metastatic therapies. However, current in vitro modeling and in vivo tracking techniques predominantly rely on fluorescent labeling to identify a limited number of relevant proteins, thereby lacking comprehensive molecular profiling at the single‐cell level.^[^ ^8^ ^]^

**Figure 1:**
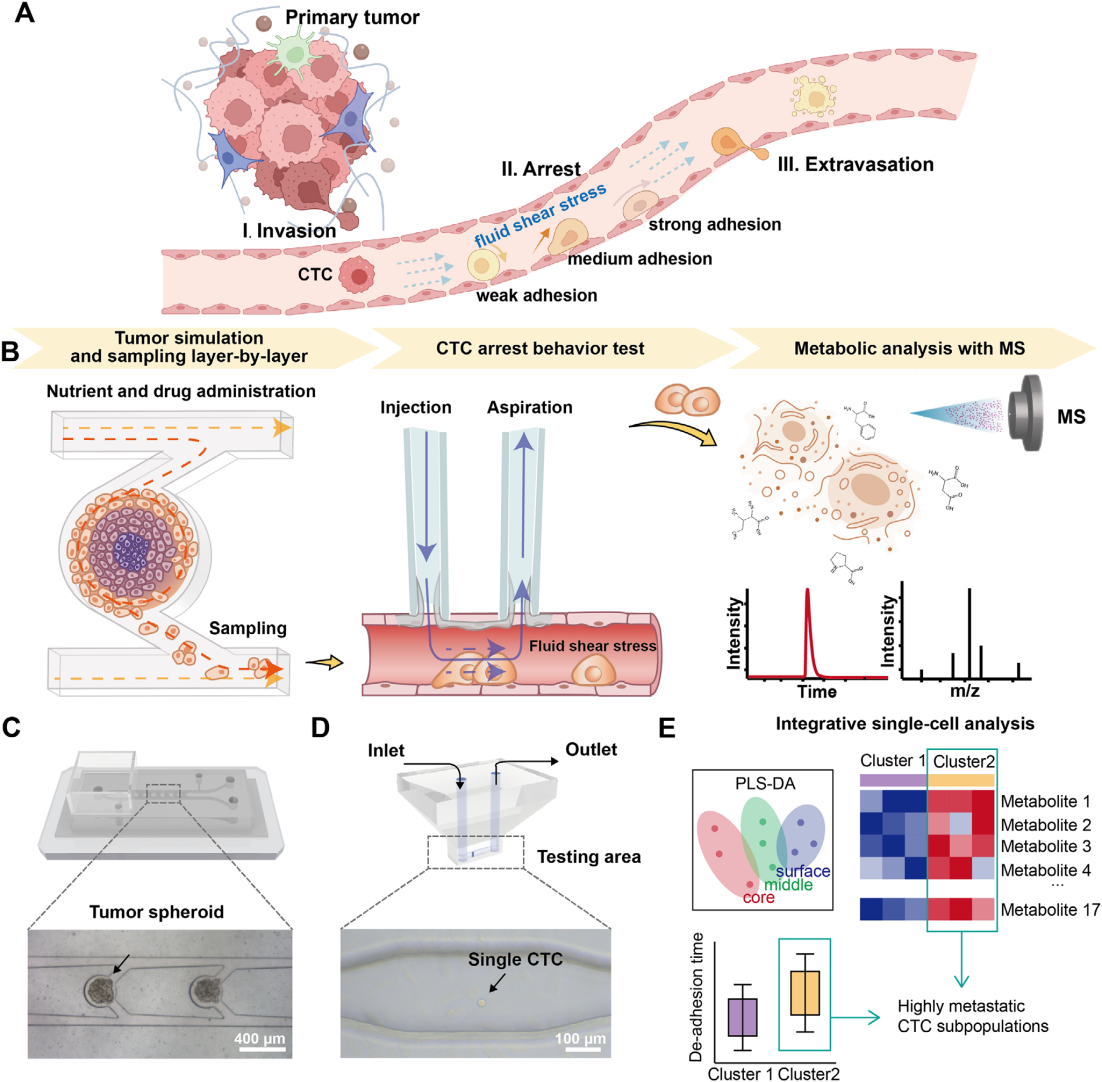
Integrated CTC behavior and metabolic analysis. A) CTCs’ arrest behavior in vasculature is heterogeneous, which plays an important role in tumor metastasis. The diagram was created with BioRender.com. B) The experimental workflow consists of a multifunctional microfluidic chip for tumor simulation and CTC sampling, a semi‐open microfluidic chip for CTC arrest behavior analysis, and MS metabolic analysis. C) Schematic diagram of the multifunctional microfluidic chip and a representative photograph of tumor spheroids cultured on the chip. D) Schematic diagram of the semi‐open microfluidic chip and a representative photograph of CTC arrest behavior test in the transient vascular system constructed by the chip and a base HUVEC cell layer. E) The integrative single‐cell analysis was performed to investigate the molecular mechanisms and potential subpopulation.

In this study, we present an integrated workflow that combines single‐cell behavioral analysis with metabolic profiling to assess the arrest behavior and metabolic heterogeneity of spheroid‐derived CTCs and their interrelationships (**Figure** **1**A,B). HCT116, a cell line with high metastatic potential and genetic heterogeneity, was used for constructing tumor metastasis models and studying CTC heterogeneity. Using a multifunctional microfluidic chip, we established standardized 3D colon cancer tumor models and categorized CTCs based on their spatial origin within the spheroid (Figure 1C). A transient vascular system was constructed with an endothelial cell layer and a semi‐open microfluidic chip, enabling surrounded fluid shear stress simulation and targeted CTC extraction (Figure 1D). The arrest capability of CTCs was quantified by de‐adhesion time, and ultra‐performance liquid chromatography‐tandem mass spectrometry (UPLC‐MS/MS) was utilized to profile critical metabolites in CTCs. Both individual CTCs and CTC clusters derived from the spheroids were analyzed, identifying metabolites correlated with their positional origin and arrest potential. Additionally, the impact of the chemotherapeutic drug 5‐fluorouracil (5‐FU) on CTC adhesion and metabolism was explored. The findings revealed the intricate interplay between CTC's arrest behavior and metabolic characteristics, underscoring the potential to identify CTCs with high metastatic propensity (Figure 1E).

## 2 Results

### 2.1 3D Tumor Modeling and Layer‐By‐Layer CTC Sampling

**Figure 2:**
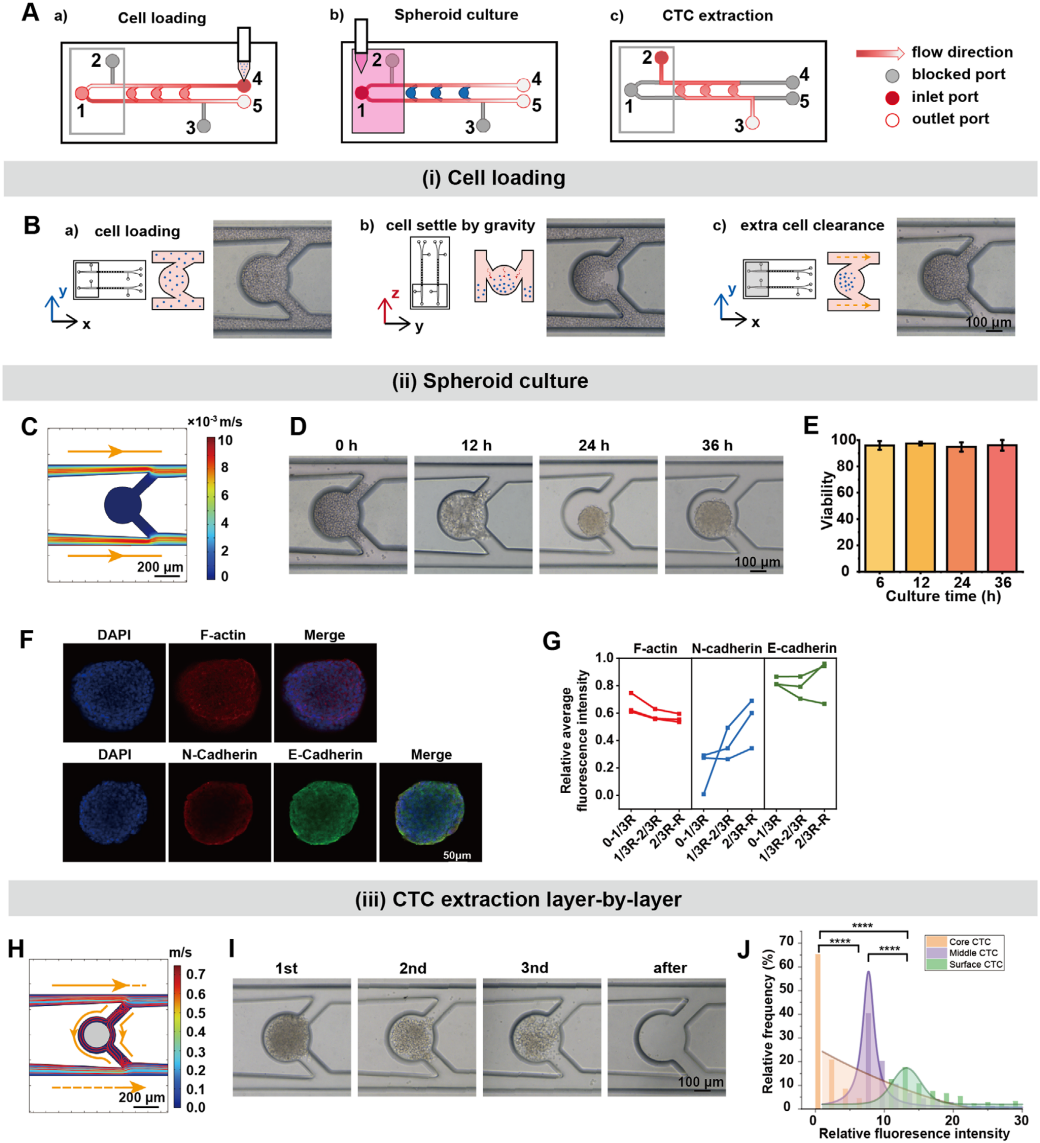
Tumor spheroid fabrication and CTC extraction on a multifunctional microfluidic chip. A) Schematic diagram showing various functions of the chip by adjusting the inlets and outlets, including a) cell loading, b) spheroid culture, and c) CTC extraction. B) Working principles and photographs of cell loading in the spheroid culture chambers. a) cell suspension loading, with all channels and chambers filled; b) cell sedimentation into the chambers under gravity; c) extra cell clearance by culture medium driven by capillary action. C) Streamline and flow rate simulation of the chip in spheroid culture mode. The medium perfusion flow rate was set at 500 µL h^−1^. D) Brightfield images recording on‐chip HCT116 spheroids formation processes. E) Cell viability of the spheroids was analyzed after 6, 12, 24, and 36 h after cell loading. (n = 13, 16, 18, 18). F) Confocal fluorescence images recording the distribution of F‐actin (red), N‐cadherin (red), and E‐cadherin (green) on the cross‐section of the spheroids. 4',6‐diamidino‐2‐phenylindole (DAPI) (blue) was used to locate the nuclei. G) The radial variation of relative fluorescence intensity in the spheroids. The average fluorescence intensity of F‐actin/E‐cadherin/N‐cadherin was divided by the average fluorescence intensity of DAPI to offset the impact of differences in dye penetration. The spheroids were segmented into three concentric layers. (n = 3) H) Streamline flow rate simulation of the chip in CTC extraction mode. The medium perfusion flow rate was set at 10 µL s^−1^. The grey circle represents the spheroid in the chamber. I) Photograph of spheroids after each digestion and the chamber after three rounds of CTC extraction. J) Statistics of the relative fluorescence intensity of CTCs extracted three times layer by layer. The spheroids were labeled with 2 µM Calcein‐AM for 15 min before the first extraction. n(surface) = 281, n(middle) = 521, n(core) = 406. (****p < 0.0001).

First, we developed a flow field‐adjustable multifunctional microfluidic chip for 3D tumor model construction and CTC acquisition. The multilayer chip consisted of a reservoir, spheroid culture chambers, a low‐cell‐adhesion polydimethylsiloxane (PDMS) film, and a glass slide, arranged sequentially from top to bottom (Figures S1A and S2, Supporting Information). Additionally, the opening status of five inlet and outlet ports of the chip could be manually controlled by acrylonitrile butadiene styrene round rods, thereby regulating the flow field distribution and enabling various functionalities of the chip (**Figure** **2**A). Specifically, ports 2 and 3 were closed during the cell loading process. A cell suspension with a concentration of 3 × 10^7^ cells mL^−1^ was introduced into the chip through port 4, and then port 5 until the channel was completely filled (Figure 2A(a), Figure 2B(a); Figure S3A, Supporting Information). The chip was then incubated vertically for 5 min, allowing the cells to deposit and occupy the spheroid culture chambers by gravity (Figure 2B(b); Figure S3B, Supporting Information). This step ensured the uniformity of the spheroids. Subsequently, the chip was placed horizontally, and little amount of cell culture medium (≈250 µL) containing 1 mg mL^−1^ Pluronic (R) F‐127 was added to the reservoir. Driven by liquid pressure, the culture medium was perfused from port 1 to ports 4 and 5 via capillary action, effectively removing excess cells from the channel (Figure 2B(c); Figure S3C, Supporting Information). As demonstrated by the hydrodynamics simulation in Figures 2C and S4A (Supporting Information), when fluid flowed through the horizontal medium supply channels, the liquid pressure generated on both sides of each culture chamber was balanced, ensuring minimal fluid passage through the chambers and preventing disturbance to the tumor cells. This principle is also employed for medium replacement during the subsequent spheroid culture process (Figure 2A(b)). With the low cell‐adhesion environment created by dissolved Pluronic (R) F‐127 and the PDMS film, cell‐cell adhesion occurred spontaneously to form a spheroid. The reservoir was filled with culture medium when cells initially adhered to each other.

During spheroid culture, the culture medium in the reservoir was replenished every 6 h, and the morphological changes of the spheroids were recorded (Figure 2D; Figure S5, Supporting Information). The initially dispersed cells gradually aggregated to form dense spheroids within 24 h, and continued to grow, nearly filling the culture chamber within 36 h. Throughout the culture process, cell viability maintained high (>90%) (Figure 2E; Figure S6, Supporting Information). As the spheroids grew, structural and microenvironmental heterogeneity akin to that of solid tumors was established,^[^ ^19^ ^]^ characterized by abundant external oxygen and nutrients and an internal hypoxic environment leading to a lower pH. Consequently, cells within the spheroids exhibited spatial heterogeneity through radial direction,^[^ ^20^, ^21^ ^]^ verified by the immunofluorescence analysis of metastasis‐related proteins F‐actin, E‐cadherin, and N‐cadherin within the spheroids (Figure 2F,G). Notably, N‐cadherin expression increased from the core to the surface, suggesting that outer cells possess greater metastatic potential, as N‐cadherin is crucial for tumor mesenchymal transformation and invasion initiation.^[^ ^22^ ^]^ Conversely, F‐actin levels slightly decreased from the core to the surface, aligning with the aggregation and cell shrinkage associated with reduced glucose supply in the spheroid core.^[^ ^23^ ^]^ E‐cadherin, indicative of cell‐cell adhesion, did not show consistent variation across the spheroid layers. However, the factors that induce tumor metastasis are diverse, and the spatial heterogeneity of tumor tissue and CTCs complicates tracing the origin of CTCs.^[^ ^24^ ^]^ Based on the assumption that CTCs are randomly shed from multiple sites on the primary tumor tissue,^[^ ^25^ ^]^ we further explored the relationship between the origin of CTCs and their metastatic potential by separating the spheroids within the chip layer by layer.

During CTC extraction, ports 1, 4, and 5 were closed, while ports 2 and 3 were opened, allowing fluid to pass through the spheroid culture chambers and generate vortex streamlines (Figure 2A(c),H; Figure S4B, Supporting Information). Changing the inlet and outlet ports also prevented residual cells in ports 1, 4, and 5 from interfering with subsequent experiments. Accutase cell digestion solution was injected via port 2, and the chip was incubated at 37 °C for 5 min. To avoid the impact of fluid streamline differences in each chamber during continuous perfusion on sampling parallelism, static dissociation was employed. Given the limited time the digestion solution acting on the spheroid, only surface loose cells detached from the spheroids were extracted. This process was repeated twice, resulting in three groups of extracted CTCs, which were classified into surface, middle, and core groups (Figure 2I; Figure S7, Supporting Information). The diffusion dynamics of Calcein‐AM in 3D tissues were used to evaluate the quality of the layered extraction.^[^ ^21^ ^]^ The fluorescence intensity of the three groups of extracted cells decreased sequentially (Figure 2J; Figure S8, Supporting Information), confirming the effectiveness of the layered extraction. The extracted CTCs and CTC clusters also maintained high viability (Figure S9, Supporting Information). When the extracted CTCs were added to a culture dish containing a HUVEC monolayer and incubated for 30 min, most cells adhered to the HUVECs and did not detach under gentle shaking, indicating their retained ability to adhere to blood vessels.

### 2.2 Vascular Microenvironment Simulation and CTC Arrest Behavior Analysis

**Figure 3:**
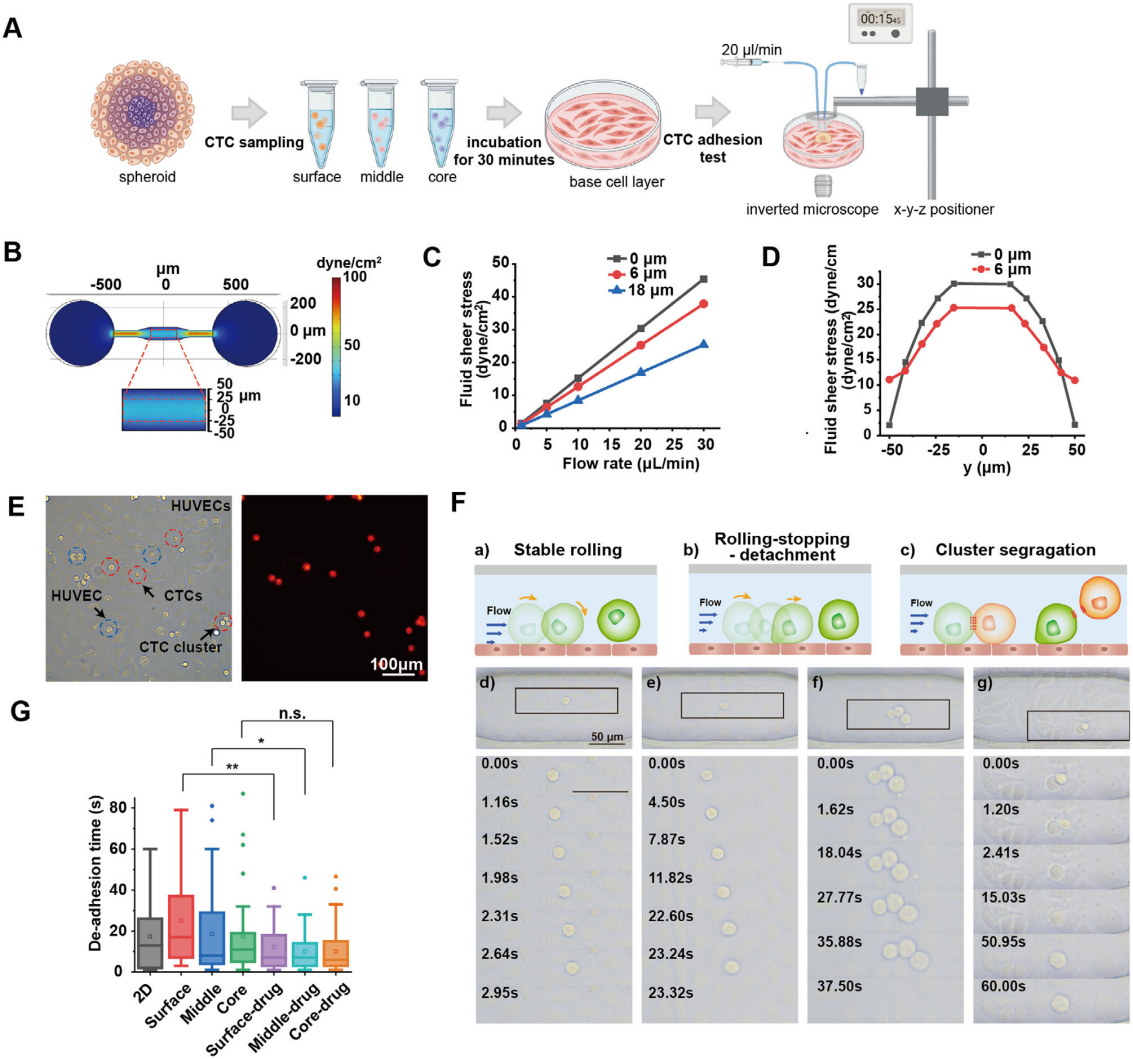
CTC arrest behavior analysis with a semi‐open microfluidic chip. A) The workflow of CTC arrest behavior analysis. The diagram was created with BioRender.com. B) Distribution of fluid shear stress simulation of the chip. The ultrapure water injection flow rate was set at 20 µL min^−1^. C) Average fluid shear stress in the detection region (−200 < x < 200 µm and −25 < y < 25 µm) at different injection flow rates. z = 0, 6, and 18 µm represent the height of the CTC‐HUVEC contact surface, the upper surface, and the multilayer CTC upper surface, respectively. D) Fluid shear stress distribution on the y‐axis in the detection region (x = 0). E) Photograph of CTCs on the HUVEC base cell layer. CTCs were labeled with CellTracker Red CMTPX during extraction. F) Schematic diagram and bright‐field image of different CTC arrest behavior under fluid shear. a,d) individual CTC stable rolling; b,e) individual CTC rolling, stopping, detachment; c,g) CTC cluster segregation; f) CTC cluster rolling, stopping, detachment. The flow rate was set at 20 µL min^−1^. G) The de‐adhesion time of CTCs samples. n = 17, 31, 35, 32, 33, 37, 32, respectively. *P* = 0.002 (Surface vs Surface‐drug), 0.03 (Middle vs Middle‐drug), and 0.06 (core vs core‐drug).

The success rate of CTCs initiating metastasis is significantly influenced by mechanical stress in blood vessels and their deformability and adhesion properties.^[^ ^26^ ^]^ Based on our previous work,^[^ ^27^, ^28^ ^]^ we developed an in‐situ CTC arrest behavioral test to analyze the extracted cell samples (**Figure** **3**A). A semi‐open microfluidic chip was designed to create a temporary vascular fluid shear environment around target CTCs and extract them simultaneously (Figures S1b and S10, Supporting Information). The chip comprised a fluid inlet channel, a target cell test area, and a fluid outlet channel. Fluid shear stress within the channel were simulated using hydrodynamics simulation (Figure 3B). The geometric center of the bottom surface of the chip is taken as the coordinate origin. The variation of average fluid shear stress with flow velocity in the region of −200 < x < 200 µm and −25 < y < 25 µm is shown in Figure 3C. It is assumed that cells exist in a hemispherical shape when adherent. Since the average diameter of HCT116 cells tested in our experiment is ≈12 µm, the fluid shear stress near the upper surface of the cells (z = 6 µm) was evaluated. At a flow rate of 20 µL min^−1^, the fluid shear stress at z = 0 µm and z = 6 µm were 30.33 and 25.27 dyne cm^−^^2^, respectively, conforming to the shear stress range of normal human arteries (10–70 dyne cm^−^^2^).^[^ ^26^ ^]^ This flow rate is also beneficial for limiting the sample volume for subsequent metabolite analyses. The y‐axis distribution of fluid shear stress in this area was also stable (Figure 3D). Therefore, a flow rate of 20 µL min^−1^ within the detection region of −200 < x < 200 µm and −25 < y < 25 µm effectively simulated the fluid environment surrounding intravascular CTCs. Notably, the shear field within the chip decreased significantly with increasing z values, at z = 18 µm (1.5 cell diameters) (Figure 3C). To eliminate interference, multilayer cells should be avoided when measuring CTC clusters.

Red fluorescently labeled CTCs were analyzed with the detection region (Figure 3E). The cells displayed heterogeneity in arrest behavior when exposed to identical fluid shear stress. Some cells exhibited a nearly uniform rolling pattern on the base cell layer until de‐adhesion (Figure 3F(a)(d); Movie S1, Supporting Information). The cell movement velocity during stable rolling (v = 2–10 µm s^−1^) was significantly lower than the fluid flow velocity at z = 6 µm (v = 0.0173 m s^−1^), indicating adhesion to the endothelial layer. This suggested that the rupture of adhesion sites between the cells and the basal cells during rolling occurred concurrently with the formation of new adhesion sites. Other cells demonstrated a rolling‐stopping‐detachment pattern when tested by the microfluidic chip (Figure 3F(b,e); Movie S2, Supporting Information). Initially, they rolled a short distance less than the cell diameter, then temporarily stopped. Under continuous fluid force, these cells underwent deformation, manifested by an increase in surface area and flattening, before finally de‐adhering. Compared to the first group, these cells likely had higher adhesion capacity and greater deformability, enabling them to establish more adhesion sites by increasing contact area with basal cells, thus enhancing their resistance to fluid shear stress. However, sustained tangential force eventually broke the receptor‐ligand interactions between the CTCs and the basal cells, leading to de‐adhesion.^[^ ^29^ ^]^ For CTC clusters, a similar rolling‐stopping‐detachment behavior was observed (Figure 3F(f); Movie S3, Supporting Information). It is noteworthy that CTC clusters may dissociate under fluid shear stress. While the overall cluster is challenged by fluid shear, the more adhesive portions could detach for stagnation and invasion (Figure 3F(c,g); Movie S4, Supporting Information). This observation may provide new insights into the greater colonization efficiency of CTC clusters.^[^ ^30^ ^]^

Inspired by similar stable rolling, stop‐and‐go, and firm adhesion behaviors described in previous numerical simulations of cell adhesion to vascular endothelium,^[^ ^31^ ^]^ we posited that stable adhesion of CTCs might occur at lower shear forces. To test this, the flow rate was reduced to 10 µL min^−1^, corresponding to a fluid shear of 12.64‐15.18 dyne cm^−^^2^, and it is observed that the cells underwent sustained deformation to nearly twice their original diameter without de‐adhering (Figure S11 and Movie S5, Supporting Information). Overall, we suggested that different behaviors exhibited by cells in experimentally simulated blood flows relate to their ability to arrest vasculature.

To quantify the arrest capacity of CTCs, the duration of adhesion for each sample was recorded. Longer residence times implied a higher likelihood of cells remaining in the vessel to complete invasion under fluid shear stress. Using this method, we assessed the arrest behavior and recorded the de‐adhesion time of 2D cultured HCT116 cells, as well as core, middle, and surface CTC groups harvested from spheroids (Figure 3G). The results demonstrated that although there was no significant difference in detachment time, spheroid‐derived CTCs exhibited more pronounced heterogeneity compared to 2D culture HCT116 cells (Figure S12, Supporting Information). Additionally, the variations in arrest ability among CTCs from different regions of the spheroids were not significant, indicating that the average results obtained by immunofluorescence do not accurately reflect the heterogeneity within the spheroids, nor can they serve as a reliable standard for judging the location and metastatic potential of CTCs. Therefore, further single‐cell molecular analysis is necessary to explore the intrinsic causes of CTC metastatic heterogeneity.

5‐FU is a broad‐spectrum antimetabolite chemotherapeutic drug that inhibits or interferes with nucleic acid synthesis^[^ ^32^ ^]^ and is the primary first‐line treatment for metastatic colorectal cancer.^[^ ^33^ ^]^ To explore the mechanism of action of 5‐FU on tumor metastasis, the CTCs from spheroids treated with 50 µM 5‐FU for 24 h were examined. After drug treatment, the arresting ability of the cells decreased except for the core group, with the most significant reduction observed in the surface group (Figure 3G). This corresponded with the structure and molecular osmotic gradient of the spheroid, where surface cells were exposed to the highest drug concentration.^[^ ^34^ ^]^ This finding suggests that 5‐FU has the potential to prevent tumor metastasis at the CTC level.

### 2.3 Integrated Behavior‐Metabolic Analysis of CTCs

For the CTCs extracted from the arrest behavior test, we assigned individual numbers to each cell and documented their classification, location, and de‐adhesion time. In total, 17 core CTCs, 17 middle CTCs, and 16 surface CTCs were recorded. Utilizing UPLC‐QQQ‐MS detection conditions we identified 17 target metabolites that could be stably detected in single‐cell samples, including aspartic acid, glutamic acid, ornithine, etc. (Table S1, Supporting Information). CTC metabolite data were analyzed using MetaboAnalyst 6.0 (https://www.metaboanalyst.ca/). Metabolite levels were log‐transformed (base 10), and data scaling was performed by mean‐centering and dividing by the standard deviation of each variable.

**Figure 4:**
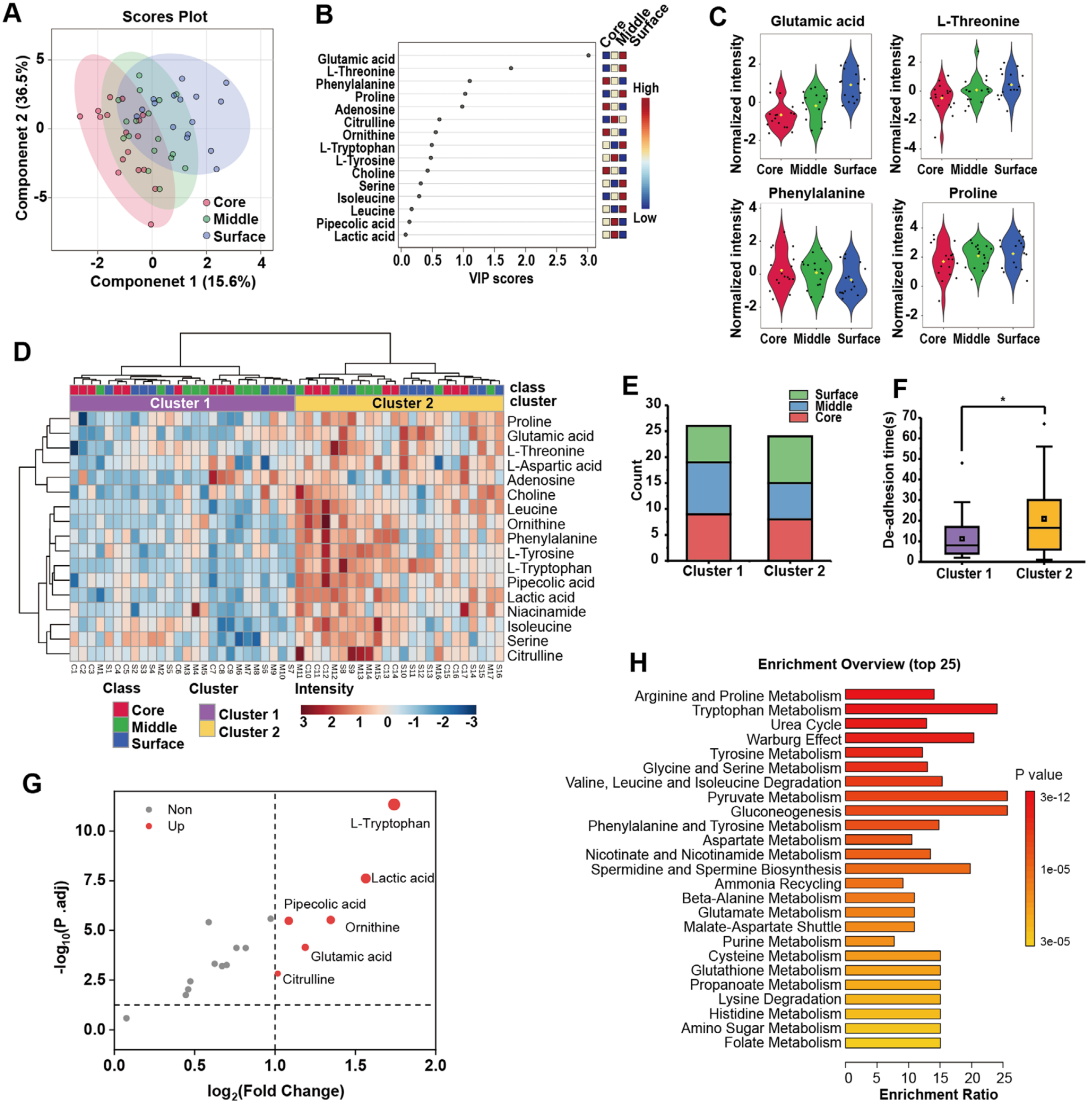
Integrated profiling of individual CTCs and identification of the subpopulations. A) PLS‐DA plot of individual CTCs from the core (n = 17), middle (n = 17), and surface (n = 16) groups. (Q2 = 0.53, R2 = 0.73, *p* < 5 × 10‐4, permutation n = 2000) B) Top 15 metabolites’ variable importance in projection (VIP) in PLS‐DA analysis. C) The relative concentration of the top four VIP score metabolites. (VIP score: Glutamic acid = 3.01; L‐threonine = 1.77; Phenylalanine = 1.10; Proline = 1.03). D) Heatmap of the 17 metabolites using Euclidean distance measures and Ward clustering. The CTCs clustered into two clusters. E) The counts of CTCs’ original group in the two clusters. F) Comparison of de‐adhesion time of the two clusters (*P* = 0.02). G) Volcano plot showing significantly different metabolites in Cluster 2 compared to Cluster 1. (fold change >2, P. adj <0.05) H) The top 25 most enriched metabolic pathways resulting from the comparison between Cluster 1 and Cluster 2.

Partial Least Squares Discriminant Analysis (PLS‐DA) was employed to cluster the normalized data. The results indicated effective separation among the core, middle, and surface groups (Q2 = 0.53, R2 = 0.73, *p* < 5 × 10^−4^, permutation n = 2000) (**Figure** **4**A). The sequentially displayed of the three groups also revealed a metabolic gradient within CTCs derived from the spheroid.^[^ ^35^ ^]^ The four most important position‐associated metabolites were glutamic acid, L‐threonine, phenylalanine, and proline (VIP scores>1.0) (Figure 4B,C). Subsequently, Euclidean distance measures and Ward clustering were applied to the samples, resulting in two clusters (Figure 4D). The proportion of cells belonging to Cluster 2 was 56.2% in the surface group, 41.2% in the middle group, and 47.1% in the core group, indicating a nearly uniform distribution across the three CTC groups within these clusters (Figure 4E). Analysis of de‐adhesion time between the two clusters showed that Cluster 2 had a significantly higher adhesion duration compared to Cluster 1 (*p* < 0.05) (Figure 4F), which may imply the existence of a subpopulation with greater arrest capacity. To further validate the presence of subpopulations and investigate differences in their actual metastatic potential, we performed dynamic cell adhesion experiments to isolate two groups of HCT116 cells with distinct adhesion abilities, defined as the Suspension group and the Adhesion group. Transwell invasion assays and wound healing experiments demonstrated that the Adhesion group exhibited significantly enhanced invasion and migration abilities. Furthermore, some cells within the Adhesion group displayed remarkably higher migration capacity compared to other cells in the wound healing assay (Figure S13, Supporting Information). Six metabolites were significantly upregulated in Cluster 2 (fold change>2, P. adj<0.05), including L‐tryptophan, lactic acid, ornithine, pipecolic acid, glutamic acid, and citrulline (Figure 4G). Enrichment analysis revealed key metabolic pathways including known amino acid metabolic pathways in tumor cells such as arginine and proline metabolism,^[^ ^36^ ^]^ tryptophan metabolism,^[^ ^37^ ^]^ as well as abnormal tumor metabolic pathways like the urea cycle^[^ ^38^ ^]^ and Warburg effect (Figure 4H).^[^ ^39^ ^]^ These findings suggested that the metabolic differences between the two clusters may be linked to variations in the arrest behavior of CTCs.

**Figure 5:**
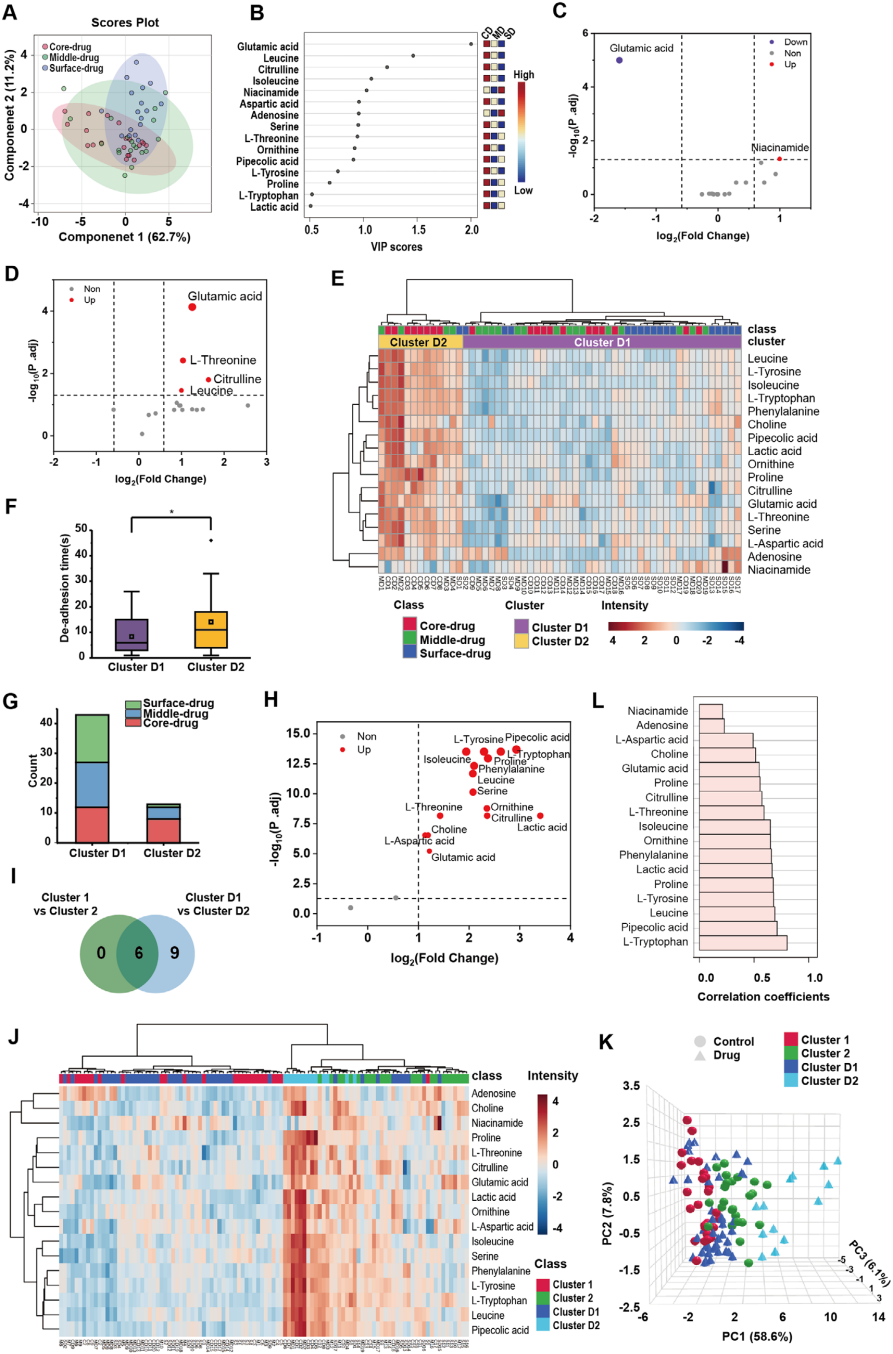
Investigation of the 5‐FU drug effect on spheroid‐derived CTCs. A) PLS‐DA plot of 5‐FU‐treated CTCs from the core‐drug (n = 20), middle‐drug (n = 19) and surface‐drug (n = 17) groups. (Q2 = 0.37, R2 = 0.66, *p* < 5 × 10^−4^, permutation n = 2000) B) Top 15 metabolites’ VIP in PLS‐DA analysis. CD refers to the Core‐drug group, MD refers to refers the Middle‐drug group, and SD refers to the Surface‐drug group. C) Volcano plot showing significantly different metabolites in surface‐drug CTCs compared to surface CTCs. (fold change >1.5, P. adj <0.05) D) Volcano plot showing significantly different metabolites in core‐drug CTCs compared to core CTCs. (fold change >1.5, P. adj <0.05) E) Heatmap of the 17 metabolites using Euclidean distance measures and Ward clustering. The CTCs extracted after drug administration clustered into two clusters. F) Comparison of de‐adhesion time of the two clusters (*P* = 0.04). G) The counts of CTCs’ original group in the two clusters. H) Volcano plot showing significantly different metabolites in Cluster D2 compared to Cluster D1. (fold change >2, P. adj <0.05) I) Venn diagram showing the intersection of the metabolites with significantly differential abundance between Cluster 2 versus Cluster 1, and Cluster D2 versus Cluster D1. J) Heatmap of all individual CTC samples with and without drug administration clustered into two clusters, with mainly Cluster 1 and Cluster D1 together, and Cluster 2 and Cluster D2 together. K) 3D interactive PCA plot of all individual CTC samples showing similar metabolic patterns between Cluster 1 and Cluster D1, as well as Cluster 2 and Cluster D2. L) Pearson r partial correlation analysis of all individual CTC samples showing the most related metabolites. Cluster 1 and Cluster D1 are combined into one cluster, and Cluster 2 and Cluster D2 are combined into the other. Position and drug administration are considered covariates of interest.

The same workflow was applied to analyze CTCs extracted after drug administration, yielding 20 core‐drug CTCs, 19 middle‐drug CTCs, and 17 surface‐drug CTCs. PLS‐DA results demonstrated a clear separation between the surface‐drug group and the core‐drug group, while the middle‐drug group exhibited partial similarity to both the surface‐drug and core‐drug group (**Figure** **5**A). This observation could be attributed to the limited drug penetration within the spheroids, affecting only the cells on the outer side of the middle layer. Five key metabolites associated with the grouping included glutamic acid, leucine, citrulline, isoleucine, and niacinamide (VIP scores>1.0) (Figure 5B).

Comparative analysis with control group CTCs revealed differential effects of 5‐FU on surface and core CTCs. Specifically, glutamic acid was significantly down‐regulated in surface CTCs, whereas niacinamide was significantly up‐regulated (Figure 5C). In contrast, four metabolites, including glutamic acid, threonine, citrulline, and leucine, were significantly up‐regulated in core CTCs (fold change>1.5, P. adj <0.05) (Figure 5D). By characterizing the penetration process of 5‐FU within the spheroid and the hypoxic conditions in its interior, supported by corresponding numerical simulations (Figure S14, Supporting Information), we propose that the differential drug responses observed in CTC groups from different spheroid regions are associated with limited drug penetration and hypoxia‐induced 5‐FU resistance.^[^ ^40^ ^]^

Similar to the control group, CTCs from the drug administration group were clustered into two categories based on metabolic patterns (Figure 5E). Cluster D1, analogous to Cluster 1, exhibited low metabolic levels and low arrest ability, while Cluster D2, similar to Cluster 2, showed high metabolic levels and high arrest ability (Figure 5F). Notably, the proportion of Cluster D2 was significantly reduced and primarily composed of core‐drug and middle‐drug CTCs (Figure 5G). In terms of metabolic characteristics, 15 metabolites were significantly up‐regulated in Cluster D2 compared to Cluster D1 (fold change>2, P. adj <0.05) (Figure 5H), encompassing the six metabolites that were up‐regulated in Cluster 2 relative to Cluster 1 (Figure 5I). This further confirmed that the CTCs derived from spheroids consist of two subpopulations with distinct differences in arrest ability and metabolism. To further verify this inference, cluster analysis and interactive PCA (iPCA) analysis were performed on all CTC samples. The results, as illustrated in Figure 5J, showed that Cluster 1 and Cluster D1 were mainly grouped into the same cluster, while Cluster 2 and C luster D2 were grouped into the other. The unsupervised 3D i‐PCA plot also showed that Cluster 1 is closer to Cluster D1, and Cluster 2 is closer to Cluster D2, confirming the existence of two subpopulations with different metabolic profiles before and after drug administration (Figure 5K). Partial correlation analysis identified several metabolites strongly associated with the clustered groups, with L‐tryptophan showing the strongest correlation (correlation = 0.80). Other strongly correlated metabolites included pipecolic acid (0.71), leucine (0.69), proline (0.68), tyrosine (0.68), lactic acid (0.66), phenylalanine (0.66), ornithine (0.65), and isoleucine (0.65) (Figure 5L). This also substantiates the metabolic distinctions between the two CTC subpopulations.

Overall, the apparent decrease in cell adhesion caused by 5‐FU was primarily due to a reduction in the proportion of highly metastatic cell subpopulations. This effect varied with the extent of 5‐FU stimulation: the number of highly metastatic CTCs in the surface group decreased by 88.9%, the middle group by 42.9%, while the core group remained almost unaffected. Additionally, the metabolism of surface CTCs showed a significant decrease in glutamic acid, which was not observed in core group CTCs, indicating a potential anti‐metastatic mechanism of 5‐FU. Glutamic acid is a metabolite generated from glutamine via Glutaminase 1 (GLS1), and elevated glutamic acid levels are considered a hallmark of metastasis in colorectal cancer patients.^[^ ^41^ ^]^ To validate the anti‐metastatic effects of 5‐FU and explore its potential mechanisms, additional experiments were performed. 5‐FU treatment was found to significantly suppress the expression of E‐cadherin and N‐cadherin in HCT116 cells. Interestingly, the supplementation of glutamic acid partially counteracted the inhibitory effect of 5‐FU on the epithelial‐mesenchymal transition marker N‐cadherin (Figure S15, Supporting Information). Dynamic cell adhesion assays further confirmed that glutamic acid supplementation alleviated the reduction in cell adhesion induced by 5‐FU (Figure S16, Supporting Information). These findings suggest that the anti‐metastatic effects of 5‐FU may be associated with the downregulation of GLS1, which disrupts glutamine metabolism and subsequently affects tumor metastasis (Figure S17, Supporting Information).

The correlation between metabolism and arrest ability of CTC clusters was further analyzed. A total of 16 core clusters (CC), 14 middle clusters (CM), 15 surface clusters (CS), 15 core clusters post‐drug (CCD), 18 middle clusters post‐drug (CMD), and 16 surface clusters post‐drug (CSD) were collected. The peak area of each metabolite was normalized by dividing by the number of cells in each CTC cluster, and the data were processed as previously described. CTC clusters from both control and drug administration groups were also grouped into two clusters, similar to the individual CTCs (Figure S18, Supporting Information). The metabolic and arrest differences between these clusters were similar to those observed in individual CTCs (Figures S19 and S20, Supporting Information). However, the difference in arrest ability between the two clusters was less obvious for CTC clusters compared to individual CTCs. Additionally, the results of cluster analysis and iPCA analysis showed less similarity between subpopulations (Figure S21, Supporting Information). This may be attributed to the metabolic heterogeneity within each CTC cluster and the more complex forces exerted on CTC clusters in the microfluidic chip simulating vascular fluid shear stress. Thus, the study of CTC clusters only provided supplementary verification.

## 3 Discussion

In this study, we developed an integrated strategy for characterizing both CTC behavior and metabolism, enabling multimodal analyses on single‐cell samples. This approach helped enhance the understanding of behavioral and metabolic heterogeneity and their interrelation in CTCs during metastasis, offering a novel method for identifying metastatic CTC subpopulations. CTCs derived from spheroids were categorized into three groups: surface, middle, and core, based on their origin within the spheroids. The arrest behavior of each CTC in a simulated vascular microenvironment, based on a semi‐open microfluidic chip, was observed and recorded. These CTCs were subsequently analyzed using integrated UPLC‐MS for metabolic profiling. Bioinformatics analysis identified two distinct subpopulations with significant metabolic and arrest ability differences. Additionally, this workflow was applied to analyze the effects of drug treatment on tumor metastasis by stimulating spheroids with 5‐FU. The results demonstrated that 5‐FU has the anti‐metastasis potential by reducing the proportion of highly metastatic clusters, manifested by decreased arrest ability and reduced glutamic acid expression. These findings suggest that our method holds potential for identifying highly metastatic CTCs and investigating anti‐metastatic drug mechanisms.

The potential of microfluidic platforms to simulate vascular microenvironments with physiological fluid flow for studying tumor metastasis has been widely discussed.^[^ ^42^ ^]^ Compared with animal models, microfluidic platforms offer low‐cost, flexible, and accessible manners to explore tumor metastasis in detail, unbound by the limitations of in vivo imaging and fluorescence imaging technologies. Previous studies have typically focused on the random movement of numerous cells under pump‐controlled shear flow,^[^ ^43^ ^]^ with few addressing the behavioral characteristics of single cells under tangential forces post‐attachment. In this work, a semi‐open microfluidic device was established to provide flexible and controllable physiological blood flow stimulation around selected CTCs while sampling cells within a defined volume. There is evidence showing that independent of passively mechanical trapping, highly metastatic cells exhibit enhanced stable arrest in larger vessels in vivo,^[^ ^29^, ^44^ ^]^ underscoring the significance of intrinsic molecular heterogeneity of CTCs in the metastatic process driven by active adhesion. Therefore, instead of focusing on CTC capture in the confined spaces of the capillary system,^[^ ^45^, ^46^ ^]^ our research analyzed the arrest behavior of CTCs against fluid shear stress without physical obstruction. Consistent with other studies, CTCs with a higher propensity for metastasis exhibited stronger adhesion to vascular endothelial cells in our in vitro vascular.^[^ ^28^, ^29^ ^]^ Existing studies on colorectal cancer metastasis have demonstrated a strong correlation between the adhesion capability of CTCs to HUVECs in vitro and their metastatic potential in vivo. CTCs enhance their adhesion to vascular endothelial cells through the upregulation of surface adhesion molecules such as EpCAM, integrins, or fibronectin receptors. This strengthened adhesion facilitates the arrest of CTCs in blood vessels and metastatic organs, significantly increasing their metastatic and invasive potential.^[^ ^47^, ^48^ ^]^ Moreover, CTCs in our study demonstrated slow rolling, arrest, adhesion strengthening, and polarization akin to those of leukocytes.^[^ ^49^ ^]^ Under fluid shear stress, CTCs with stronger arrest ability increased their contact area with endothelial cells through deformation, thereby stabilizing the arrest process. This observation is consistent with previous studies, which reported that enhanced deformability of CTCs directly correlates with increased invasiveness.^[^ ^50^ ^]^ These results suggested that CTCs with high metastatic potential can be identified through their behavioral phenotypes. There is evidence indicating that fluid shear stress within a certain range promotes CTC colonization in vivo.^[^ ^44^ ^]^ However, in vivo or clinical studies have rarely elucidated the behavioral dynamics of CTCs on vascular walls under fluid shear stress or the correlation between CTC deformability and invasiveness. These limitations are primarily due to the fragility of CTCs and the challenges associated with real‐time tracking in vivo.^[^ ^51^ ^]^ In contrast, our study successfully visualized and tracked CTC arrest behaviors by simulating a vascular microenvironment around single cells in vitro, combined with real‐time microscopic observation. Notably, our observations consist of existing computational predictions,^[^ ^31^, ^52^ ^]^ demonstrating the utility of our approach in bridging experimental and theoretical insights into CTC behavior.

CTCs carry information about the heterogeneity of primary tumors and adjust their metabolic composition to adapt to microenvironmental changes during metastasis. Consequently, the molecular characteristics of tumor cells have become crucial biomarkers and therapeutic targets.^[^ ^53^ ^]^ Single molecular diagnostic technologies may overlook rare subclones with metastatic potential.^[^ ^5^ ^]^ Thus, the joint evaluation of single CTCs is key to addressing this issue. In our work, CTCs with high metastatic tendency were identified, characterized by high expression of six metabolites, all of which play key roles in tumor metastasis. For instance, elevated lactic acid levels in clinical samples are positively correlated with metastasis in various cancers such as colorectal cancer,^[^ ^54^ ^]^ melanoma,^[^ ^55^ ^]^ and gastric cancer.^[^ ^56^ ^]^ Lactic acid accumulation reflects increased energy demand for tumor progression, promotes tumor angiogenesis and metastasis, and mediates immune escape.^[^ ^57^ ^]^ Glutamic acid, produced by glutamine breakdown, indicates heightened metabolic demand in tumor cells. Upregulated expression of GLS1 and the cystine antiporter xCT, involved in glutamic acid production and transport, is associated with recurrence, invasion, and lymph node metastasis in colon cancer.^[^ ^41^, ^58^ ^]^ The transport of tryptophan, ornithine, and citrulline within tumors corresponds with their depletion in the plasma of metastatic cancer patients, suggesting that tumor cells promote metastasis through the L‐arginine metabolic pathway and the tryptophan‐kynurenine pathway.^[^ ^59^, ^60^, ^61^, ^62^ ^]^ Pipecolic acid has also been linked to tumorigenesis.^[^ ^63^ ^]^ Moreover, all six metabolites detected in our study have been identified in serum, fecal, or tissue metabolomics analyses of colorectal cancer patients at different disease stages.^[^ ^64^ ^]^ Among them, lactic acid, glutamic acid, and tryptophan levels in tumor tissues increase exponentially with disease progression from Stage I to Stage IV, reflecting metastatic progression within patients.^[^ ^65^, ^66^ ^]^ Meanwhile, citrulline, ornithine, and pipecolic acid are significantly elevated in the serum of colorectal cancer patients compared to healthy controls.^[^ ^67^, ^68^ ^]^ These findings confirm that the metabolite signatures detected in our study are consistent with the metabolic profiles observed in clinical colorectal cancer samples and correlate with disease progression. Most current studies on tumor metastasis metabolism rely on large tumor tissue samples or enriched CTCs, which overlook the intrinsic heterogeneity within CTC populations. Advances in single‐cell metabolomics have the ability to reveal the existence of subpopulations within CTCs and explore their differences from primary tumor tissues.^[^ ^69^, ^70^ ^]^ For example, using metabolomics screening and machine learning approaches, a metabolic fingerprint comprising four metabolites associated with metastasis was identified in colorectal cancer cell lines with varying metastatic abilities. This fingerprint was successfully applied to identify high‐metastatic CTC subpopulations from colorectal cancer patients, with elevated levels of glutamic acid and lactic acid consistent with our results.^[^ ^71^ ^]^ By directly correlating single‐cell metabolic analyses with the arrest behaviors of individual CTCs, our approach allows for the identification of metabolic features associated with CTC metastatic potential and corresponding drug responses. This method reduces reliance on extensive data processing and prior knowledge, providing a more straightforward pathway for understanding the link between CTC metabolism and metastasis.

To investigate the relationship between the origin of CTCs and their metastatic potential, we employed various methods. Immunofluorescence staining provided insight into the molecular heterogeneity within spheroids, however it only reflected average information. The CTC arrest behavior test highlighted the heterogeneity of metastatic potential at the single‐cell level, but the statistical analysis did not obtain a significant result. The integrated workflow, which associated the arrest phenotypes and metabolic profiles of individual CTCs, offered a multimodal tool to identify CTC subpopulations with high metastatic potential and elucidate the metabolic factors underlying CTC heterogeneity. This analysis demonstrated that the source of heterogeneity lies in the distribution differences of highly metastatic subpopulations within spheroids. Using spheroid models and drug stimulation, the capability of this method to analyze complex tissue and microenvironment changes was validated. Additionally, this workflow is highly scalable. The spheroid culture device holds the potential to simulate more complex tumor microenvironments. For instance, medium supply channels can mimic the interstitial flow of the tumor,^[^ ^72^ ^]^ and co‐culturing tumor cells with other cell types, such as fibroblasts and immune cells, in the cell culture chamber can create a heterogeneous cellular condition more representative of the clinical tumor microenvironment.^[^ ^73^, ^74^ ^]^ Tumor organoids could also be constructed in the chamber by patient‐derived tumor cells dispersed in Matrigel and supplemented with growth factors.^[^ ^75^ ^]^ Besides, a more comprehensive understanding of CTC metastasis may be achievable by integrating our technology with more advanced single‐cell metabolic detection technologies, such as single‐cell RNA sequencing and high‐resolution mass spectrometry.

This workflow still has several restrictions. First, the throughput requirements of single‐cell detection are unneglectable. The operation time for single‐cell arrest testing and extraction is ≈2–3 min per cell, posing a considerable labor burden when detecting large sample sizes. Developing automated equipment for the simultaneous detection of multiple samples may address this issue. Second, to better replicate the in vivo behavior of CTCs within blood vessels, the physiological relevance of the vascular microenvironment in the current microfluidic system needs further improvement. For instance, microfluidic chips with diverse structures, such as bifurcations and varying channel widths, could be developed to simulate complex vascular networks. Additionally, seeding HUVECs onto PDMS surfaces could mimic the elastic properties of blood vessels. Simultaneously, perfusion fluids supplemented with sodium carboxymethyl cellulose and polystyrene microspheres could mimic the rheological and cellular properties of blood while remaining compatible with downstream metabolic analyses. Finally, while this method demonstrates the capability to analyze CTC clusters, the current approach of measuring deadhesion time and averaging the metabolism of each cell in the cluster remains overly simplistic. Given the crucial role of CTC clusters in metastasis, a more detailed analysis of clusters will be a key focus for our future work. Specifically, high‐speed cameras could be employed to track the deformation and motion trajectories of individual cells within CTC clusters, while fluorescence probes could be used to characterize molecular tension between cells.^[^ ^76^ ^]^ Furthermore, computational simulations based on discrete element methods (DEM) and finite element methods (FEM) could provide detailed insights into cell‐cell interactions within the clusters and the overall mechanical forces acting on the clusters.

In conclusion, the integrated single‐cell analysis of CTC arrest behavior and metabolism established in this study demonstrates a capability to identify CTCs with high metastatic potential and evaluate CTC drug responses. Liquid biopsy based on CTC testing is gaining prominence as a method for cancer screening and monitoring, where CTC counts commonly serve as biomarkers. Increasing evidence underscores the importance of CTC heterogeneity, highlighting the need to identify specific CTC subpopulations that are capable of initiating metastasis. Functionally integrated single‐cell multimodal analysis can uncover molecular information related to specific phenotypes from a distinctive perspective. Given the unique role of CTCs in the metastasis process, the approach shows great potential in addressing this challenge. With further optimization of detection accuracy and physiological relevance, it holds promise as an important tool to support clinical diagnostics and predictive methodologies.

## 4 Experimental Section

### Microfluidic Devices Fabrication

The patterns of the two microfluidic devices were designed with Adobe Illustrator 2020. First, the silicon wafer‐positive mold was made with SU‐8 2050 negative photoresists and developer (Microchem Corporation, USA) based on its processing guidelines (www.kayakuAM.com). The finished silicon molds were silanized by 1H,1H,2H,2H‐perfluorooctyl trichlorosilane (Macklin, China) overnight. Then the microfluidic chips were made of PDMS (Dow Corning, USA) by soft lithography. The height of the microchannels was 300 µm for the tumor model construction chip and 100 µm for the vascular simulation chip. The detailed scale of each device is shown in Figure S1 (Supporting Information). The PDMS with microstructures were then bonded onto glass slides or other PDMS slides using an air plasma cleaner (Harrick, USA).

### Cell Culture

HCT116 and HUVEC were purchased from the Cancer Institute and Hospital (Chinese Academy of Medical Science, China). All cells were cultured in Dulbecco's modified eagle medium (DMEM) (Solarbio, China) supplemented with 10% fetal bovine serum (FBS) (Gibco Corporation, USA), 100 µg mL^−1^ penicillin (Solarbio, China), and 100 µg mL^−1^ streptomycin (Solarbio, China). The cells were maintained at 37 °C in a humidified atmosphere of 5% CO_2_. All cells were recovered every 2–3 days to keep subconfluently using 0.25% trypsin with 0.02% EDTA (Solarbio, China).

### Microfluidic Chip Pretreatment

The tumor model construction microfluidic chips needed pretreatment before cell seeding. The chips were hydrophobically modified by 1H,1H,2H,2H‐perfluorooctyl trichlorosilane overnight. Immersed the chips in 10 mg mL^−1^ Pluronic (R) F‐127 (Sigma‐Aldrich, USA) aqueous solution in the vacuum dryer to remove gas inside the chip and then incubated for 2 h for low adherence of the PDMS film. The chips were sterilized by UV irradiation for 30 min. Before filling with cells, the liquids inside the channels were replaced with phosphate buffer solution (PBS) (Solarbio, China) three times, and finally replaced with culture medium. Gas should not be introduced into the channel during the operation.

### On‐Chip Cell Culture

The chips carrying tumor spheroids were maintained in the Heracell 250i incubator (Thermo Scientific, USA) at 37 °C in 5% CO_2_. The culture medium of DMEM supplemented with 1mg mL^−1^ Pluronic (R) F‐127, 10% FBS, 100 µg mL^−1^ penicillin, and 100 µg mL^−1^ streptomycin was added into the reservoir every 6 h. During the culture, ports 4 and 5 were sealed with a few drops of culture medium to prevent drying, and PBS was added to the culture dish containing the chip to prevent evaporation of the liquid within the chip. A Leica DMi8 inverted fluorescence microscope (Leica, Germany) was used to observe and record the morphological changes of the spheroids.

For hypoxia experiments, the culture medium was supplemented with 1 µM Image‐iT Green Hypoxia (Thermo Scientific, USA). After 36 h of incubation, cross‐sectional images of the spheroids were captured using a Zeiss LSM780 inverted confocal laser scanning microscope (Zeiss, Germany). The images were analyzed with ImageJ software.

### Spheroids Staining

To evaluate the cell viability of the spheroids, ports 1, 4, and 5 were closed, and PBS containing 2 µM Calcein‐AM and 3 µM PI (Dojindo Laboratories, Japan) was injected through port 3. The spheroids were incubated for 30 min at 37 °C. The cross‐sectional spheroid fluorescence images were captured using a Zeiss LSM780 inverted confocal laser scanning microscope, and the images were analyzed using ImageJ software. Cell activity was calculated by dividing the green fluorescence intensity by the total fluorescence intensity. The results were expressed as means ± standard errors.

For immunostaining of the spheroids. Replaced the culture medium with PBS three times. The spheroids were fixed in 4% (v/v) paraformaldehyde in physiological saline for 20 min. After each of the steps that follow, wash the chips with PBS three times. The spheroids were permeabilized with 0.5% (v/v) Triton X‐100 for 30 min, and then incubated in a blocking buffer (Beyotime, China) for 20 min. Next, filled the chip with 1 µg mL^−1^ E‐cadherin monoclonal antibody (rabbit) and 1 µg mL^−1^ N‐cadherin monoclonal antibody (mouse) (Beyotime, China) at 4 °C for at least 12 h. After washing with PBS, replaced with Alexa Fluor 647‐labeled F(ab″)_2_‐goat anti‐mouse IgG (H+L) secondary antibody and Alexa Fluor 488‐labeled F(ab″)_2_‐goat anti‐rabbit IgG (H+L) secondary antibody (Beyotime, China) at a dilution ratio of 1:500 and incubated at 4 °C for 2–3 h in the dark. For F‐actin evaluation, TRITC Phalloindin (Solarbio, China) was diluted to 100 nM, and incubated at room 4 °C for 30 min. Finally, the nuclei were stained for 5 min using the DAPI staining solution. The cross‐sectional fluorescence images of spheroids were captured using a Zeiss LSM780 inverted confocal laser scanning microscope, and the images were analyzed using ImageJ software. The cross‐sectional images were segmented into three concentric layers using Photoshop. Specifically, the fluorescence image contours were first labeled, followed by sequential radial equal scaling of the contours to 1/3 and 2/3. The average fluorescence intensity of each layer was examined. The average fluorescence intensity of F‐actin/E‐cadherin/N‐cadherin was then divided by the average fluorescence intensity of DAPI to offset the impact of differences in dye penetration.

For drug penetration simulation experiments, Rhodamine 6G (Macklin, China) was used as an alternative to visualize 5‐FU molecule penetration within the spheroid. Spheroids were cultured for 36 h, and then the medium was supplemented with 5 µM Rhodamine 6G. Cross‐sectional fluorescence images of the spheroids were captured using a Zeiss LSM780 inverted confocal laser scanning microscope at time points of 1, 2, 4, 8, and 24 h. The images were analyzed using ImageJ software.

### CTC Extraction

Following three PBS washes, Accutase cell digestion solution (Yeasen, USA) containing 10 µM CellTracker Red CMTPX (Thermo Fisher Scientific, USA) was injected via port 2, and the chip was incubated at 37 °C for 5 min. The digestion solution was then removed with PBS, and the chip was gently vibrated to disengage loose cells. Detached cells were extracted by injecting PBS. This process was repeated twice, resulting in three groups of extracted CTCs. The red fluorescent marker was used to distinguish the CTCs from the base cell layer in subsequent experiments.

### CTC Arrest Testing Device Setup

For the sample preparation of CTC arrest testing, 5 × 10^5^ HUVEC cells were seeded into a 10 cm diameter Petri dish and cultured for 48 h until a dense cell layer was formed as the base cell layer. Before planting the CTCs, the medium was washed with PBS three times and then replaced by Dulbecco's phosphate‐buffered solution (D‐PBS) with Ca^2+^ and Mg^2+^ (Solarbio, China) containing 0.025 M 4‐ (2‐hydroxyerhyl) piperazine‐1‐erhanesulfonic acid (HEPES) solution (Solarbio, China). The extracted CTCs were added to the base cell layer and incubated for 30 min before testing. The arrest testing experiments were performed on a Leica DMI 4000B inverted fluorescence microscope (Leica, Germany). The semi‐open microfluidic chip was fixed on an XYZ positioner (Sigma KOKI, Japan), and the positioner was adjusted until the bottom surface of the chip was parallel to the bottom of the Petri dish. The inlet of the chip was connected to a syringe filled with ultrapure water, and the outlet of the chip was connected to a 500 µL tube for sample collection. The positioner was controlled to point the detection region of the chip at randomly selected CTCs, and then the chip was slowly lowered until a closed analyzing space was formed. A syringe pump (LSP02‐2B, Longer Pump, China) was turned on to inject ultrapure water at 20 µL min^−1^, while the video software of the microscope recorded the CTC arrest behavior. The injection continued for 30s after cell de‐adhesion to ensure the cell transferred into the sample tube. Isovolumetric acetonitrile (Honeywell, USA) was promptly added to each sample for processing.

### LC‐MS/MS Analysis

HCT116 cells were harvested and washed with ultrapure water three times. The cell suspensions were then diluted to 50 cells mL^−1^, mixed with acetonitrile at a ratio of 1:1, and used as the sample for condition optimization. The metabolites were screened for stable detection and the mass spectrometry detection was optimized. A Shimadzu Cellent CM‐MS with LCMS‐8050 (Japan) was used to quantify the metabolite concentration in the culture medium. A CAPCELL PAK ADME‐HR column (2.1mm i.d.×75 mm) pack with 3 µm adamantane particles was used in UPLC. The elution mobile phase was (A) 0.1% formic acid in ultrapure water and (B) 0.1% formic acid in acetonitrile. The flow rate maintains at 0.30 mL min^−1^, with phase A at 0.2700 ml/min and phase B at 0.03 mL min^−1^. 1 µL sample for a single test and three parallel tests for each sample. The mass spectrometric instrument was as follows: the interface temperature was 300 °C, the heating block temperature was 250 °C, the desolation line was 150 °C, the spray voltage was 6kV, the nebulizer gas (N_2_) flow rate was 3 L min^−1^, the heating gas (N_2_) and drying gas (N_2_) flow rate was 10 L min^−1^. The multiple reaction monitoring (MRM) mode parameters of cell metabolites are presented in Table S1 (Supporting Information). The CTC samples were subsequently analyzed under optimized mass spectrometry conditions, and the peak areas of the target metabolites in each sample were relatively quantified using the instrument software.

### Statistical Analysis

All numerical results were expressed as the mean ± standard error of at least three independent experiments and statistically compared using two‐way analysis of variance (ANOVA) and unpaired two‐tailed t‐test. The p‐values for significance testing were set at 0.05 (*), 0.01 (**), 0.001(***), 0.0001(****), and n.s. indicates not significant. For any multiple comparison analysis, p‐values were adjusted using the Benjamini‐Hochberg method to control the false discovery rate. Scientific illustrations were generated by Origin2021.

### Bioinformatics Analysis

Metaboanalyst (version 6.0) was used to analyze the CTC metabolite and de‐adhesion data. The data normalization of metabolite levels was produced by log‐transformed (base 10), then data scaling by mean‐centering and dividing by the standard deviation of each variable. The “Statistical Analysis [one factor]” module was used to analyze the metabolic profile of CTCs. Dimensional reduction was performed by Partial Least Squares Discriminant Analysis (PLS‐DA). Each group of CTCs was divided into two clusters under the Euclidean distance measures and Ward clustering. The differential metabolites were identified using the unpaired two‐tailed t‐test with the threshold of Fold change>2.0 and P. adj <0.05, p‐values corrected by the Benjamini‐Hochberg method (FDR = 0.05). The “Statistical Analysis [metadata table factor]” module was used to perform partial correlation analysis and unsupervised 3D i‐PCA. The quantitative enrichment analysis was also developed with Metaboanalyst 6.0.

## Conflict of Interest

The authors declare no conflict of interest.

## Supporting information



Supporting Information

Supplemental Movie 1

Supplemental Movie 2

Supplemental Movie 3

Supplemental Movie 4

Supplemental Movie 5

## Data Availability

The data that support the findings of this study are available from the corresponding author upon reasonable request.
