# Supplementary material for: Linking Metastatic Behavior and Metabolic Heterogeneity of Circulating Tumor Cells at Single‐Cell Level Using an Integrative Microfluidic System
Source: Adv Sci (Weinh). 2025 Feb 17;12(14):2413978. doi: 10.1002/advs.202413978 (PMC11984876; doi:10.1002/advs.202413978)
Supplement: Supplementary file 1 — Supporting Information [file ADVS-12-2413978-s006.docx]

Supporting Information

Linking Metastatic Behavior and Metabolic Heterogeneity of Circulating Tumor Cells at Single-Cell Level using an Integrative Microfluidic System

Ying Hou, Jiaxu Lin, Hongren Yao, Zengnan Wu, Yongning Lin, Jin-Ming Lin*

Department of Chemistry, Beijing Key Laboratory of Microanalytical Methods and Instrumentation, Key Laboratory of Bioorganic Phosphorus Chemistry & Chemical Biology (Ministry of Education), Tsinghua University, Beijing 100084, China
E-mail: jmlin@mail.tsinghua.edu.cn.

**Immunofluorescence staining**

Cells were seeded at a density of 1.5 × 10⁴ cells per well in a 96-well plate and cultured at 37°C in a 5% CO₂ incubator for 24 h. After incubation, the medium was replaced with DMEM complete medium containing 5 μM 5-FU, 5 μM 5-FU combined with 1 mM glutamic acid, or DMEM complete medium for the control group. Following a 24-hour treatment, immunofluorescence staining was performed according to the procedures described in the "Spheroids Staining" section of the Experimental Section. The expression levels of E-cadherin, N-cadherin, Phospho-Histone H2A.X (Ser139), and Glutaminase (GLS) were characterized in each group. The primary antibodies used were Glutaminase monoclonal antibody (rabbit) and Phospho-Histone H2A.X (Ser139) monoclonal antibody (mouse), both purchased from MedChemExpress (USA). Fluorescence images were captured using a Zeiss LSM780 inverted confocal laser scanning microscope (Zeiss, Germany), and image analysis was performed using ImageJ software.

**Dynamic cell adhesion assay**

HUVEC cells were seeded in a 6-well plate and cultured for 48 h to form a monolayer. Adherent HCT116 cells were treated with 5 μM 5-FU, 5 μM 5-FU combined with 1 mM glutamic acid, or DMEM complete medium for the control group. After treatment, the HCT116 cells were harvested and labeled with CellTracker Red CMTPX. The labeled HCT116 cells were washed and added to the 6-well plate containing the HUVEC monolayer at a density of 6 × 10⁴ cells per well. The plate was incubated at 37°C with gentle shaking at 90 rpm for 30 min. Unattached cells were removed by washing the wells with PBS. Fluorescence images were captured using a Leica DMi8 inverted fluorescence microscope (Leica, Germany), and the images were analyzed using ImageJ software to quantify the number of adhered cells per field of view.

**Transwell invasion assay**

HCT116 cells were serum-starved in DMEM medium for 12 h, harvested, and resuspended. The cells were then seeded onto a 100 mm culture dish pre-coated with a monolayer of HUVEC cells. The dish was incubated at 37°C with gentle shaking at 90 rpm for 30 min. The non-adherent cells in the supernatant were collected as the Suspension group. The adherent cells attached to the HUVEC layer were harvested as the Adhesion group.

In a 24-well plate equipped with PET Transwell chambers (8 µm pore size, LABSELECT, China), 100 µL of Matrigel Matrix (Corning, USA) diluted 1:8 in serum-free DMEM was added to each upper chamber. The plate was incubated at 37°C for 2 h, and excess liquid was carefully removed. Subsequently, 100 µL of serum-free DMEM was added to each chamber and allowed to gel hydration at 37°C for 30 minutes.

Then the two groups of HCT116 cells were resuspended at a density of 2 × 10⁶ cells/mL in serum-free DMEM. A total of 100 µL of each cell suspension was seeded into the upper chamber of the Transwell. The lower chamber was filled with 600 µL of DMEM supplemented with 20% FBS as a chemoattractant. The plate was incubated at 37°C in a humidified atmosphere with 5% CO₂ for 24 h.

Following incubation, the Transwell chambers were fixed in 4% (v/v) paraformaldehyde for 15 minutes. The chambers were washed with PBS and stained with 0.1% crystal violet dye (Solarbio, China) for 15 min. After staining, the chambers were washed three times with PBS, and the cells remaining on the upper side of the membrane were carefully removed using a cotton swab. Images were captured using a Leica DMi8 inverted fluorescence microscope (Leica, Germany). The number of invasive cells per field of view was quantified using ImageJ software.

**Wound-healing assay**

HCT116 cells from the Suspension group and Adhesion group were seeded into 6-well plates at a density of 5×10⁵ cells per well and cultured in complete DMEM until the confluence reached 90%. Wounds were created using a pipette tip. After washing the cells three times with PBS, the medium was replaced with DMEM containing 2% FBS. Images were captured at 0 h and 24 h using a Leica DMi8 inverted fluorescence microscope, and the migration distance was analyzed using ImageJ software.

**Numerical simulation**

The penetration process of the drug within the tumor spheroid was simulated using the Michaelis-Menten kinetics model. The simplified model incorporates the following processes and conditions: the drug diffuses from the surrounding culture medium into the tumor spheroid, while cells absorb the drug at a specific uptake rate, resulting in a concentration gradient within the spheroid. Specifically, the dynamics of drug concentration within the tumor spheroid are described by the following governing equation:^[1]^

$$\frac{\partial c}{\partial t}+\nabla\cdot(-D\nabla c)=-\left( N_{\text{cell }}\frac{V_{max}c}{K_{m}+c} \right)$$

Where *c* represents the drug concentration; *D* represents the diffusion coefficient of the drug in the culture medium or within tumor cells; *N*_cell_ represents the cell density; $V_{max}$ represents the maximum drug uptake rate by cells; *K*_m_ is the Michaelis-Menten constant.

The same model was used in the simulation of the oxygen concentration within the spheroids. Specific simulation parameters are provided in Table S2. Finite element analysis was conducted using commercial simulation software (COMSOL Multiphysics, version 6.1).


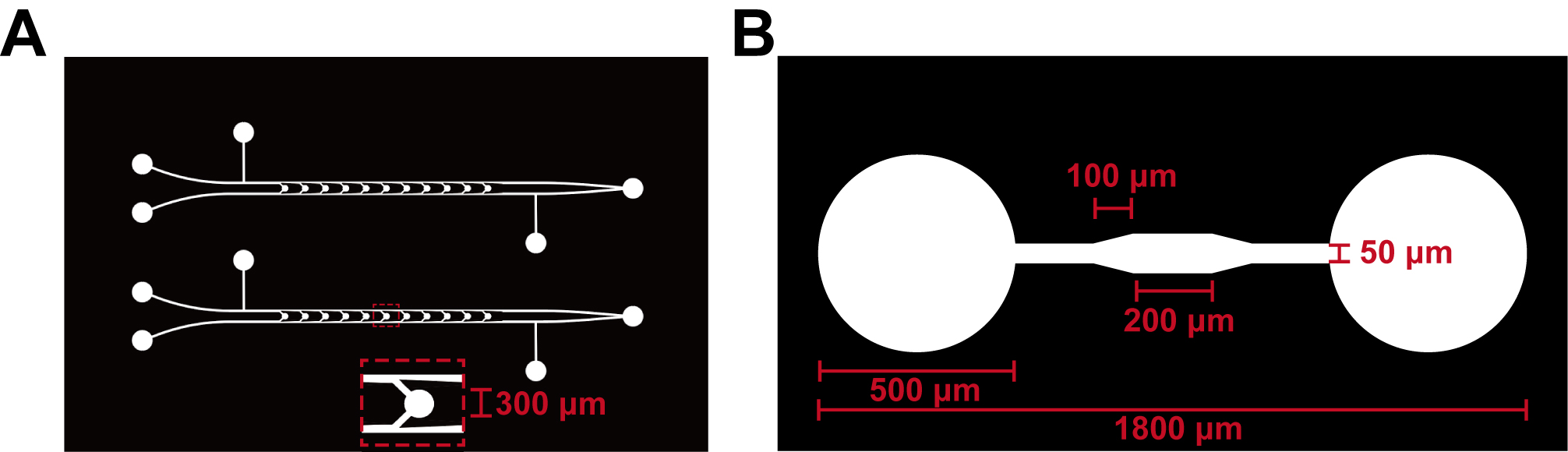


**Figure S1**. The pattern of the masks shows the structure and detailed parameters of (A) the multifunctional microfluidic chip and (B) the semi-open microfluidic chip.


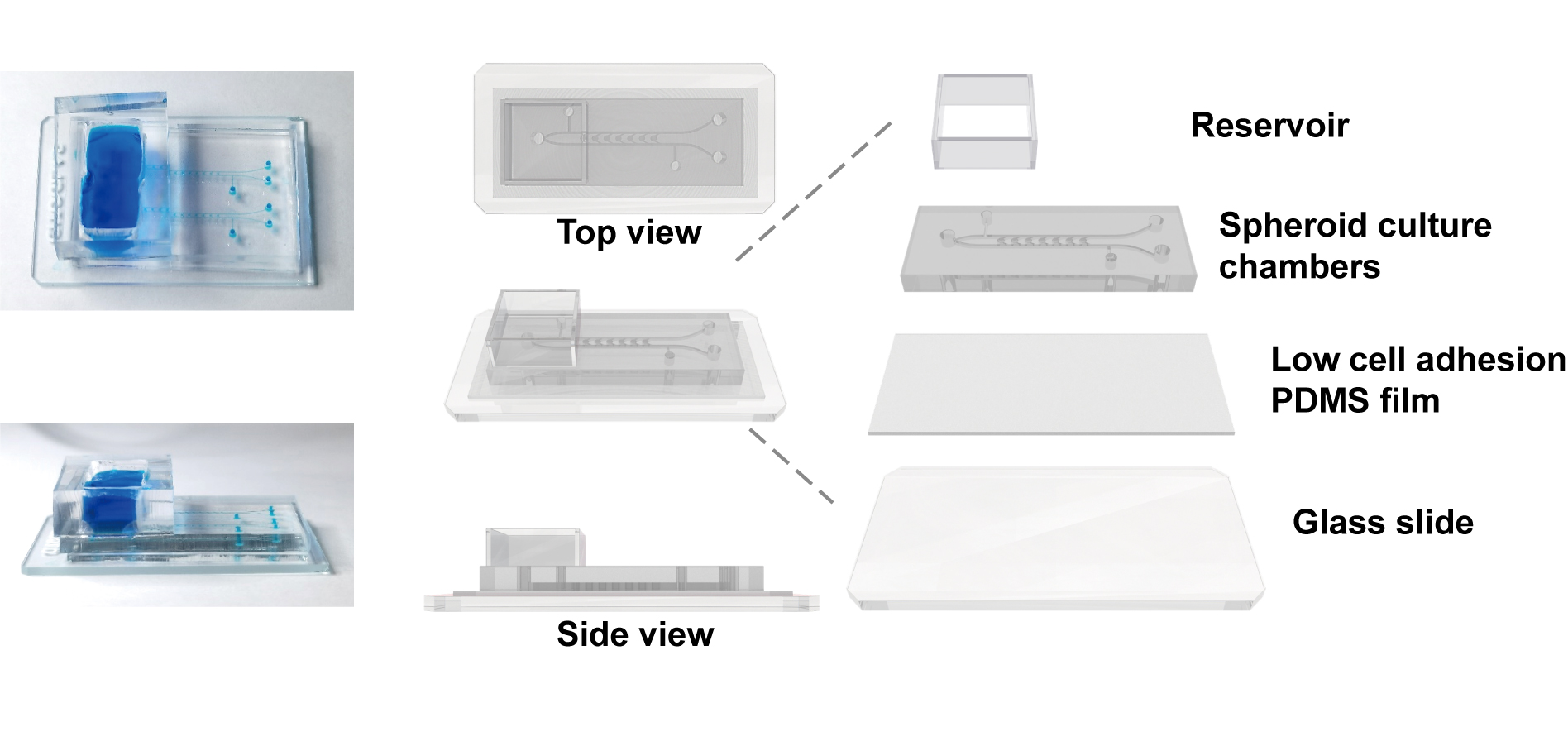


**Figure S2.** Schematic diagrams and photographs of the multifunctional microfluidic chip for tumor simulation and CTC sampling.


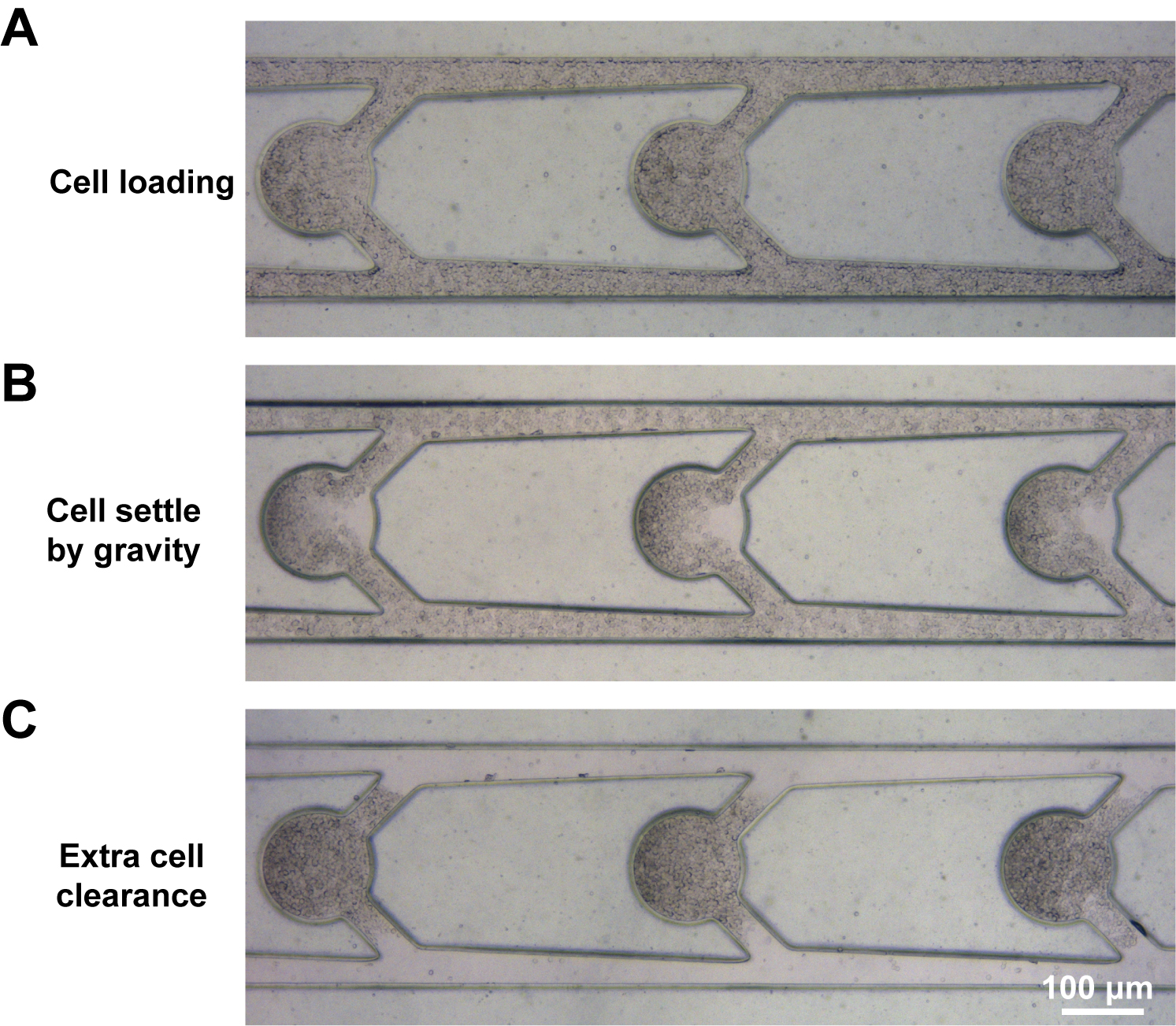


**Figure S3**. Photographs of cell loading in three successive spheroid culture chambers. (A) cell suspension loading, with all channels and chambers filled; (B) cell sedimentation into the chambers under gravity; (C) extra cell clearance by culture medium driven by capillary action.


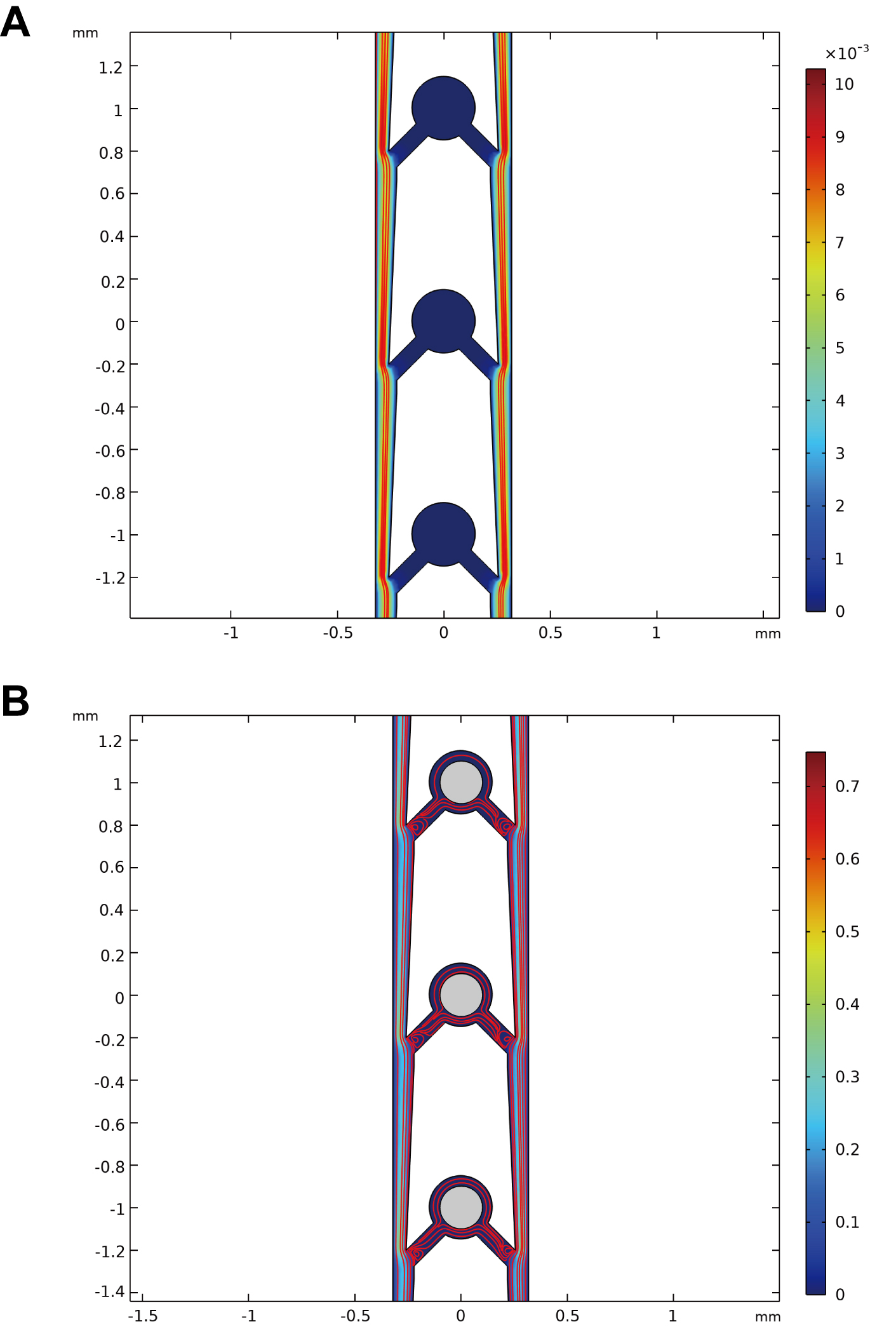


**Figure S4**. Streamline and flow rate simulation of three successive spheroid culture chambers of the chip in (A) spheroid culture mode and (B) CTC extraction mode. The medium perfusion flow rate was set at 500 μL/h and 10 μL/s, respectively. The grey circles represent the spheroid in the chamber.


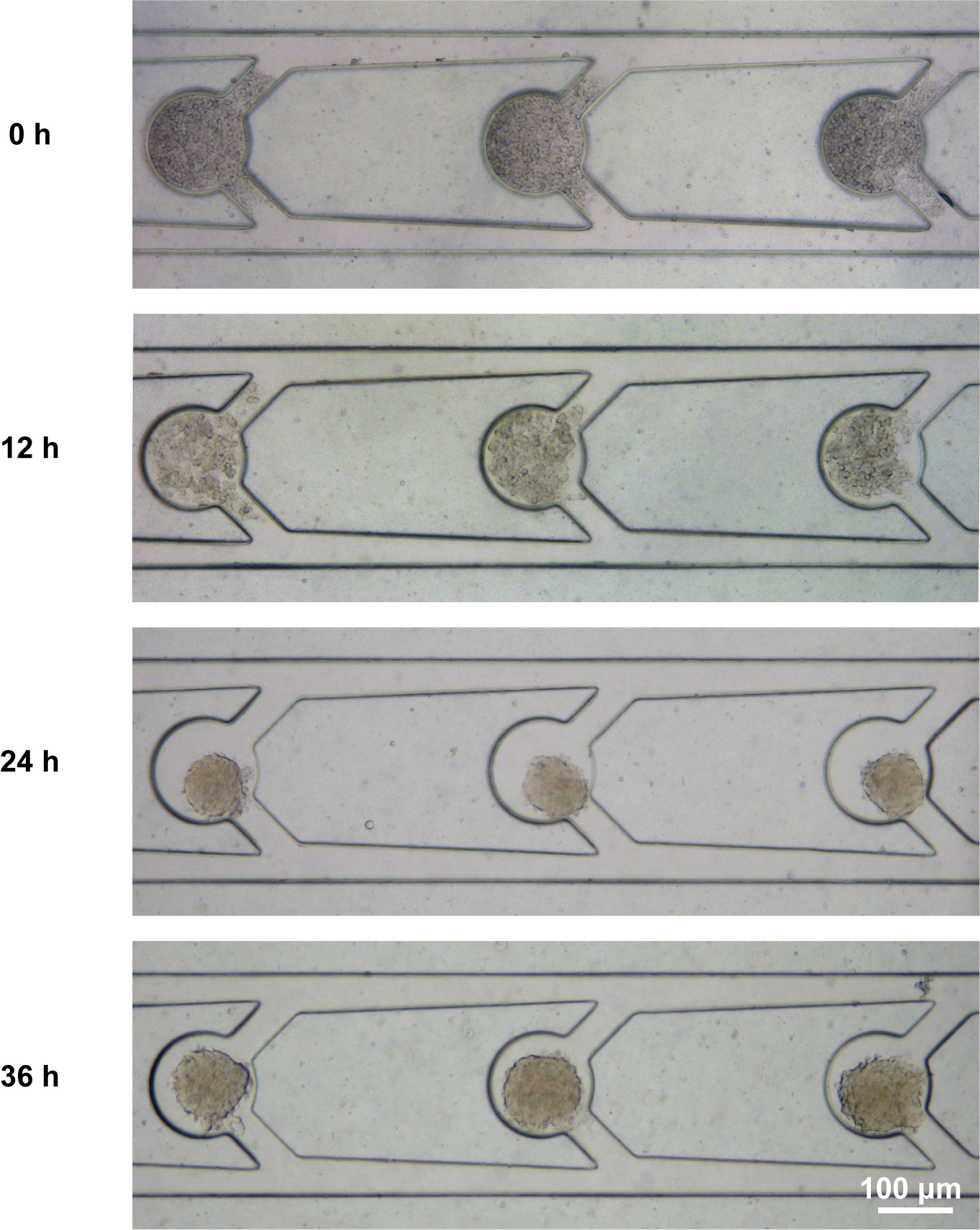


**Figure S5**. Brightfield images recording on-chip HCT116 spheroids formation processes in three successive spheroid culture chambers, indicating the consistency of spheroid formation.


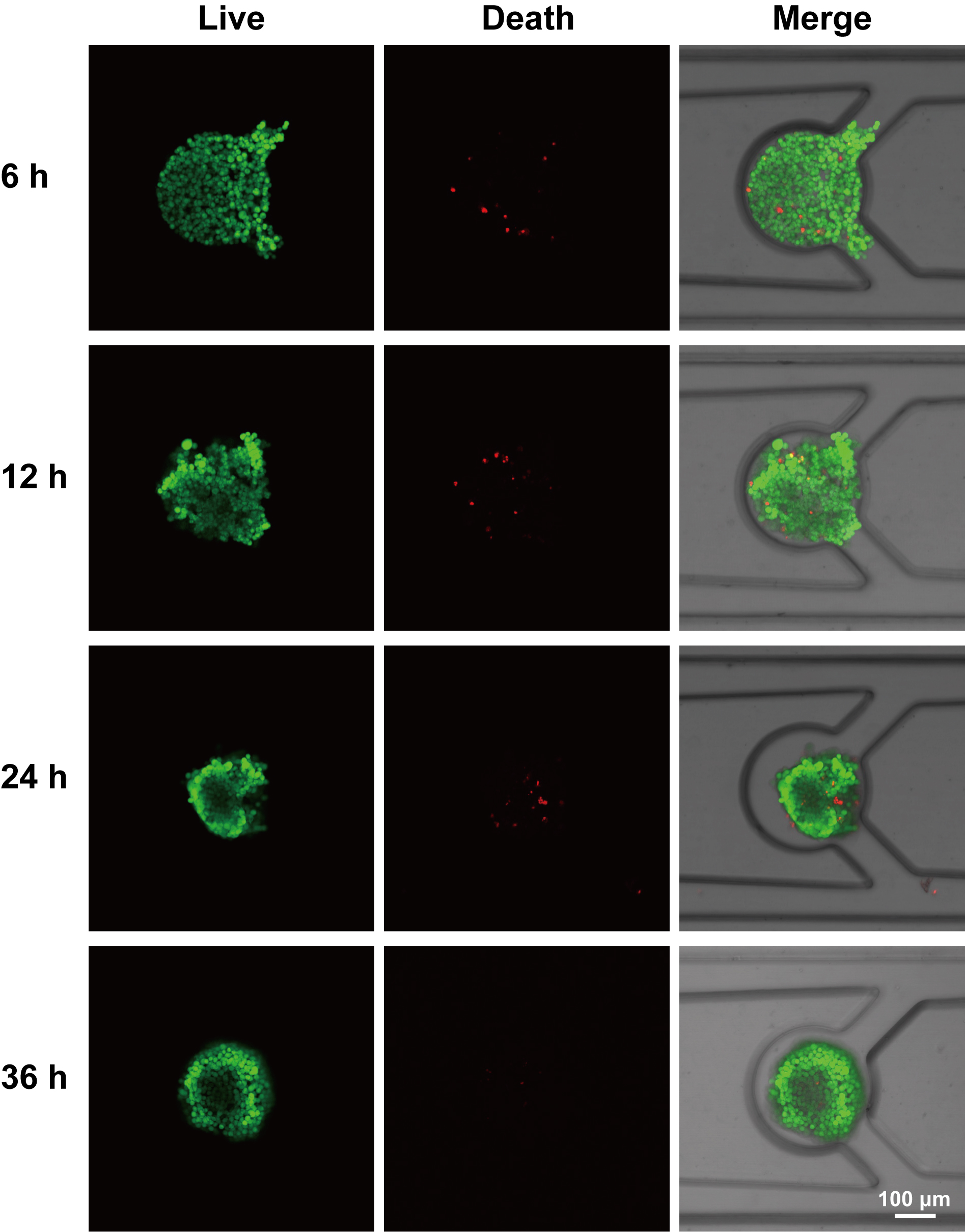


**Figure S6**. Fluorescence images of spheroids stained with Calcein AM/PI during the culture periods, showing the viability of the cells.


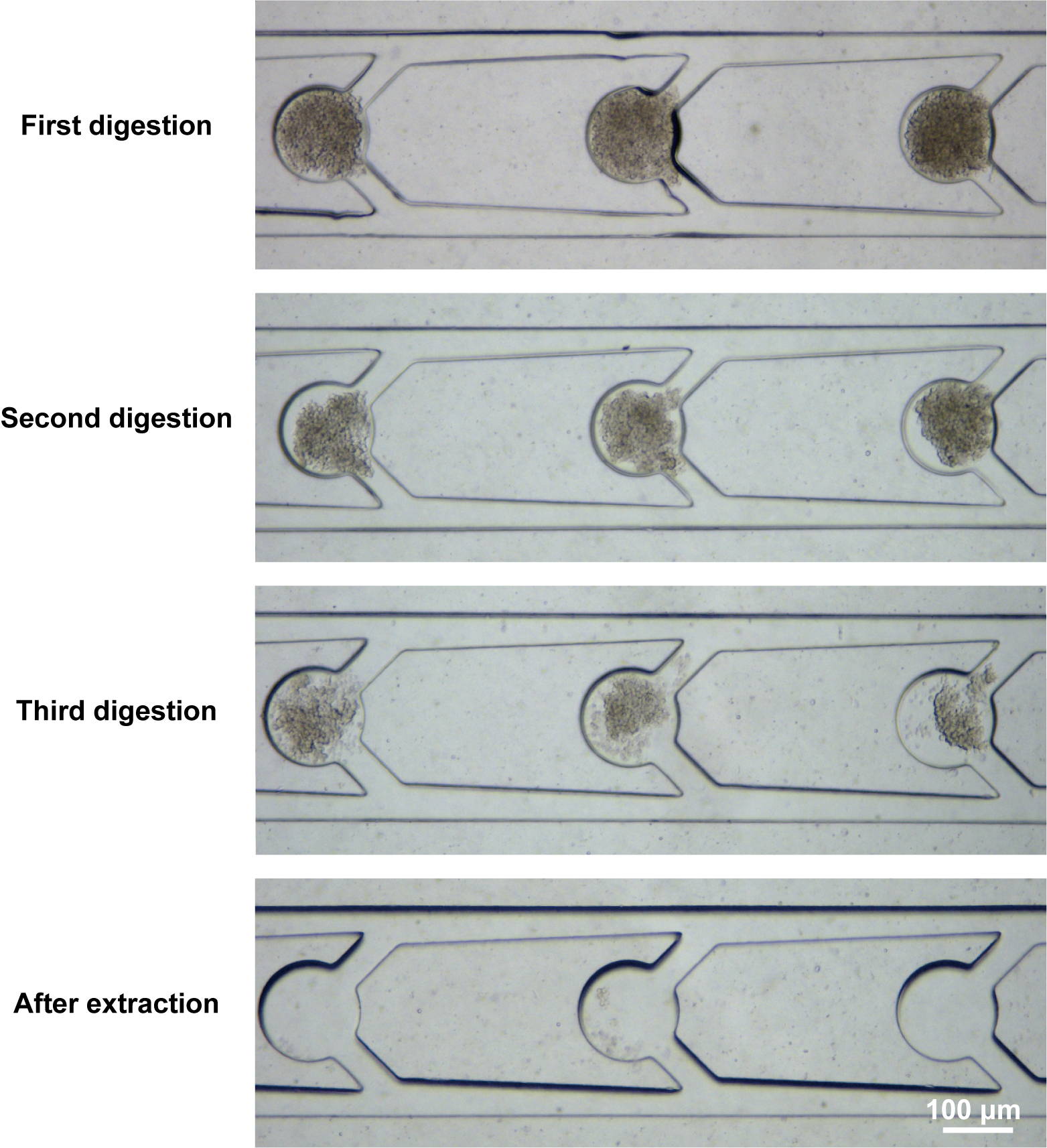


**Figure S7**. Brightfield images of spheroids in three successive spheroid culture chambers after each digestion and the chamber after three rounds of CTC extraction, indicating the consistency of the CTC extraction process.


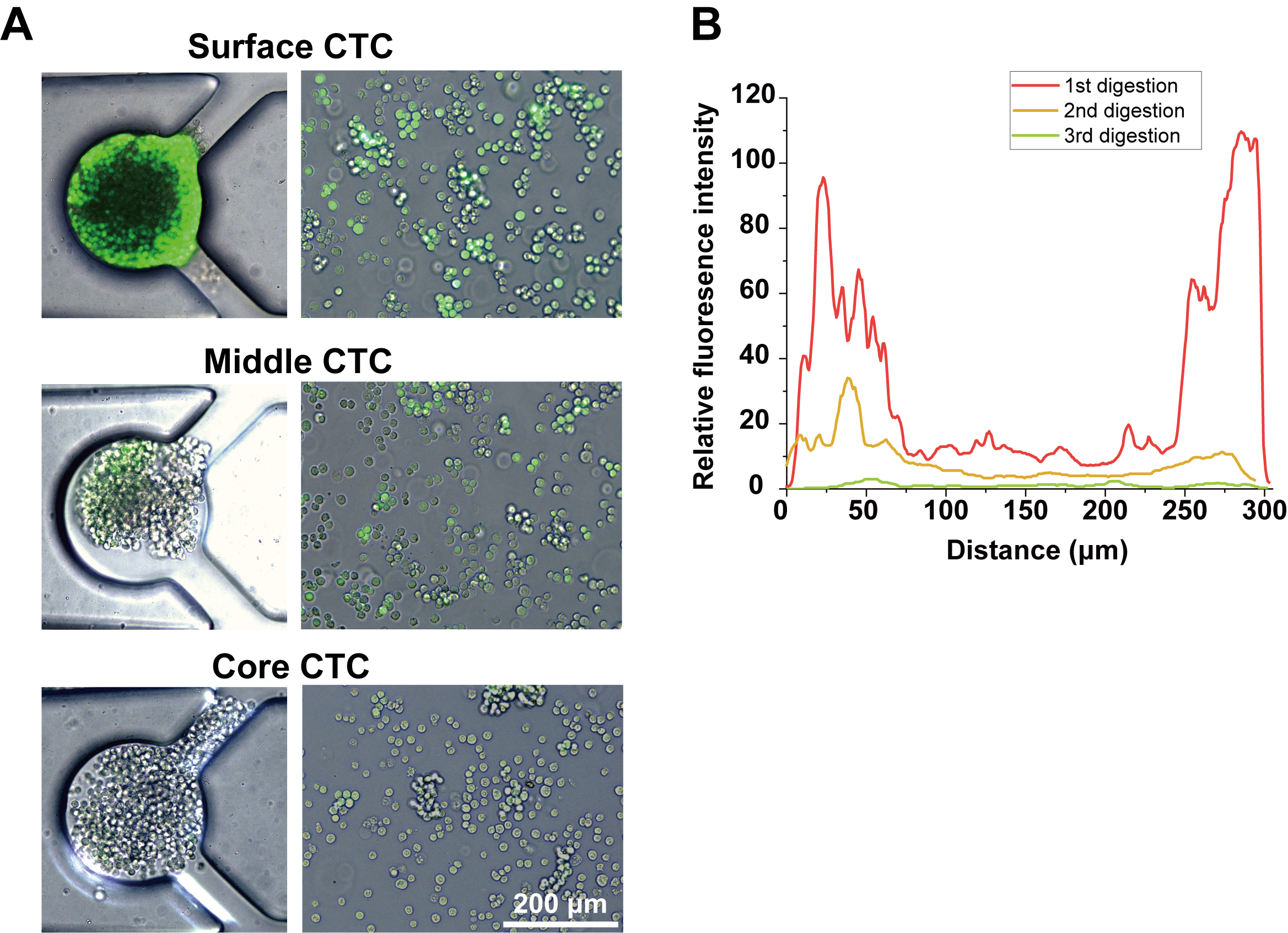


**Figure S8.** Effectiveness evaluation of the layered extraction of CTCs. (A) Merge of bright field and fluorescent images showing the spheroids after three times of digestions, and the recovered CTCs. The spheroids were labeled with 2 μM Calcein-AM for 15 min before the first extraction. Green fluorescence of CTCs indicated their original place in the spheroid. (B) Fluorescence intensity distribution on the radial direction of cells within the chamber after three times of digestion.


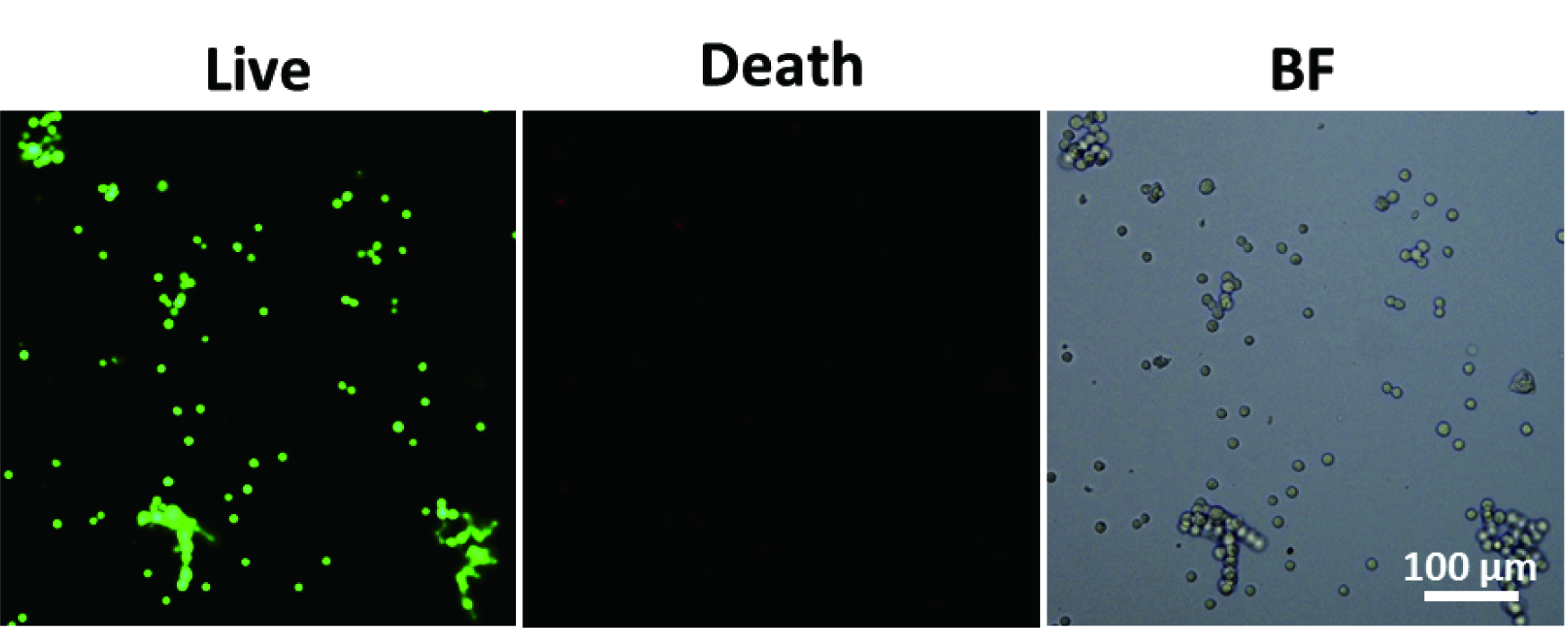


**Figure S9**. Fluorescence images of CTCs extracted from the spheroids. Calcein Am/PI staining shows the viability of the cells.


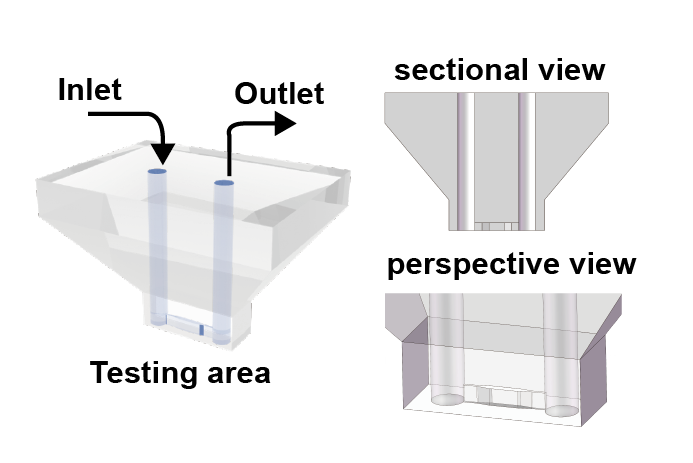


**Figure S10**. Schematic diagram of the semi-open microfluidic chip with the sectional and perspective views showing the inner structure.


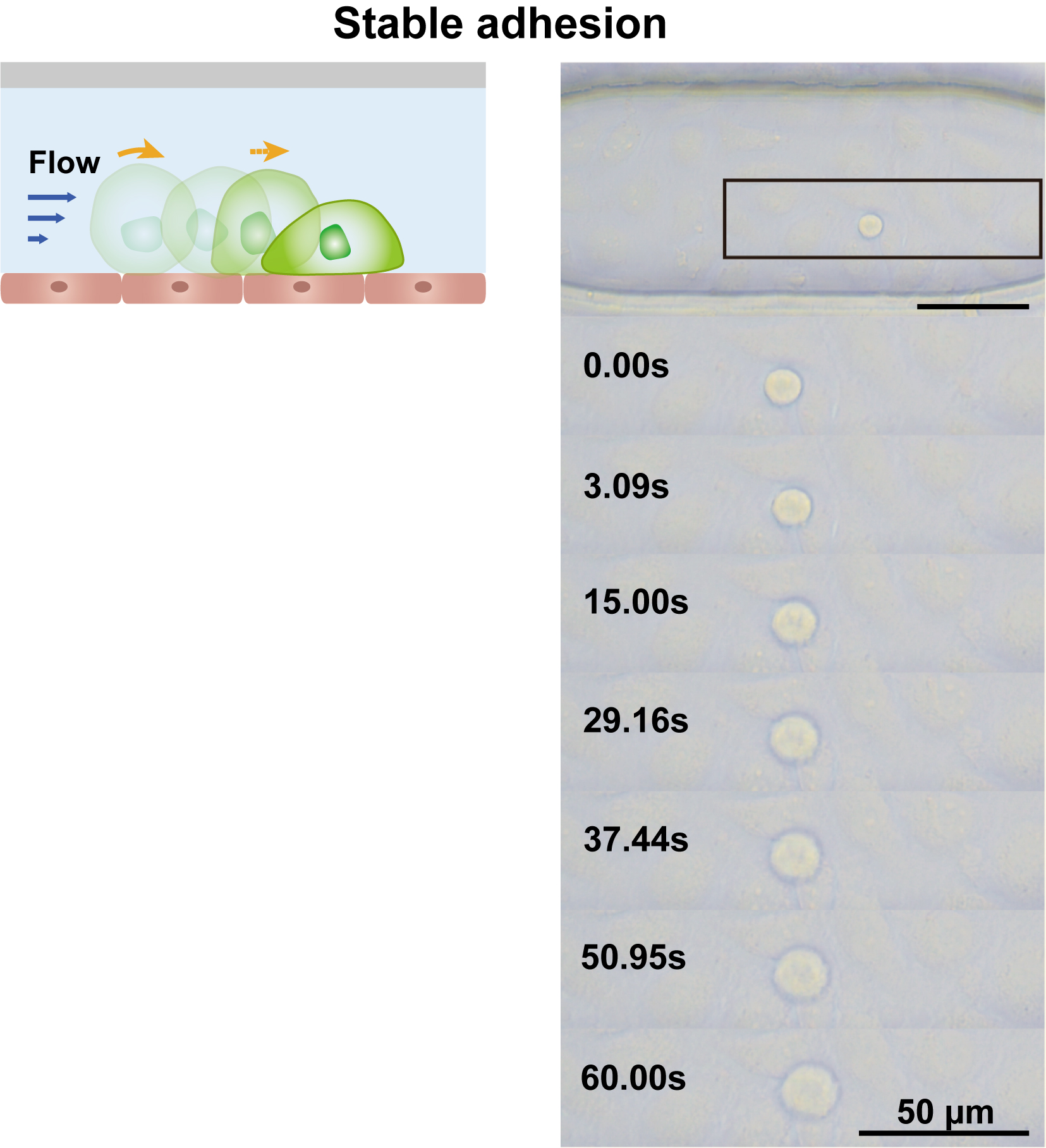


**Figure S11.** Schematic diagram and bright-field image of CTC showing stable adhesion behavior under fluid shear stress. The flow rate was set at 10 μL/min.


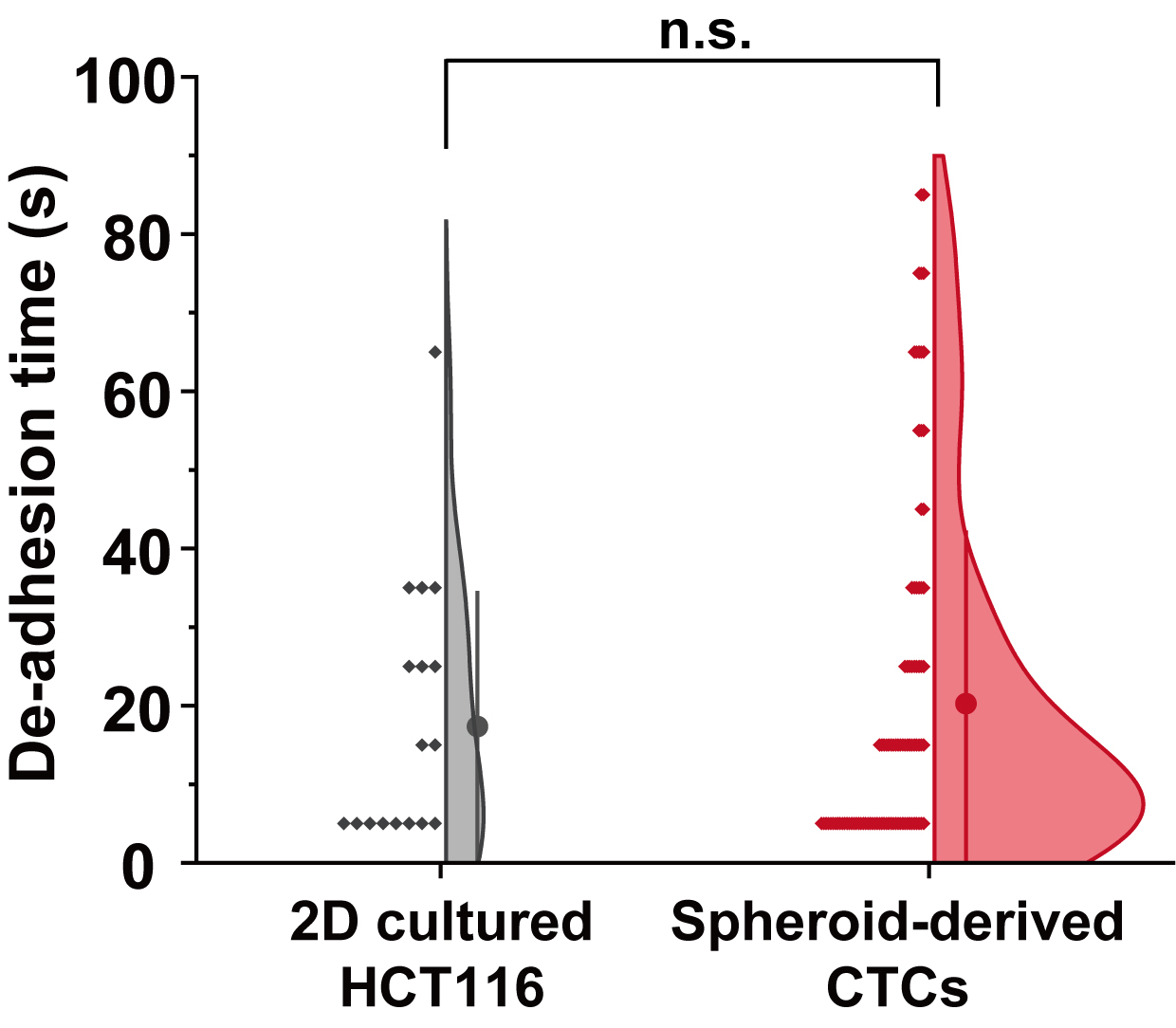


**Figure S12**. Violin plots showing the de-adhesion time distribution of 2D culture HCT116 cells and spheroid-derived CTCs in the arrest behavior test. n = 17 and 98.


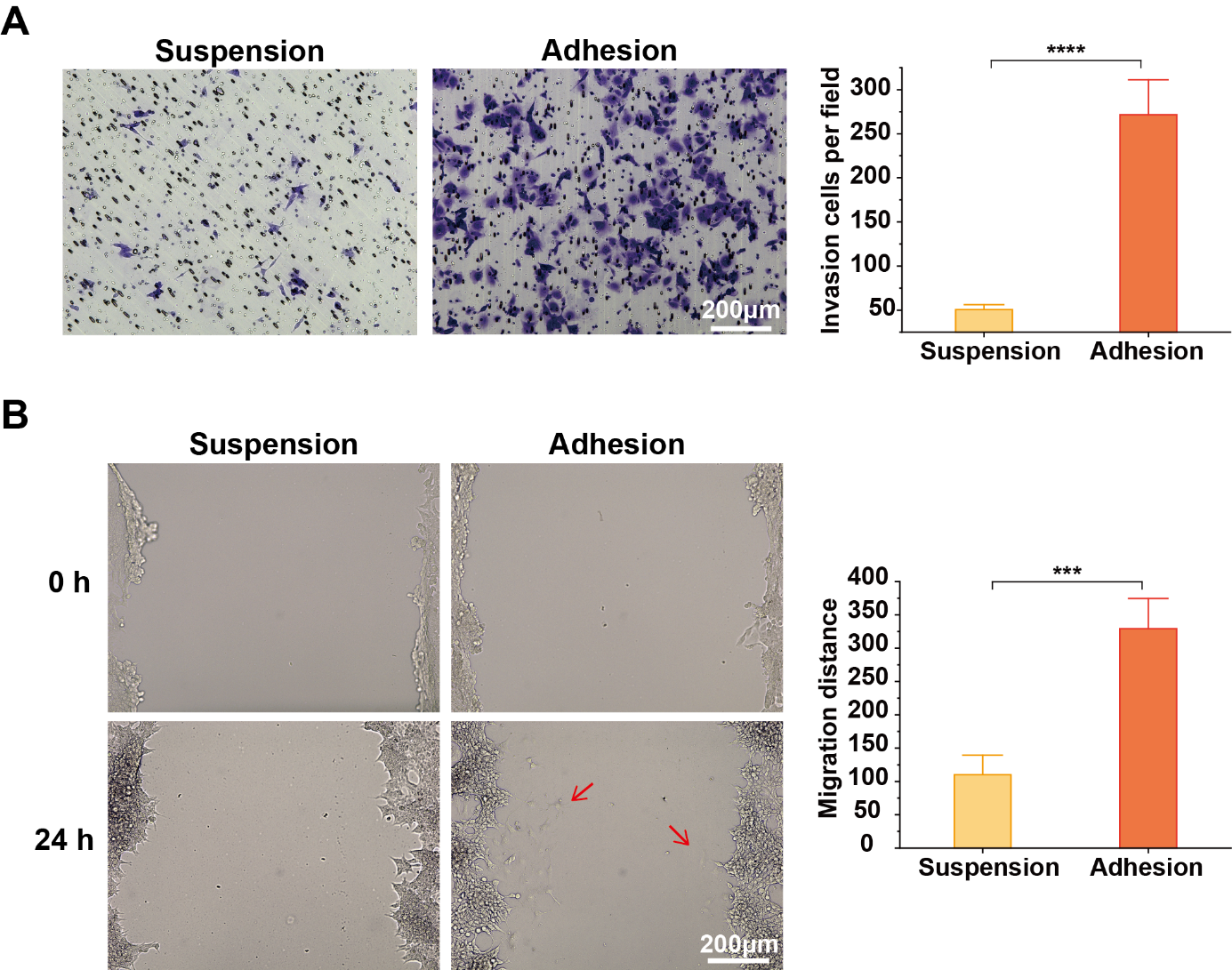


**Figure S13**. (A) The comparison of Transwell invasion assay of Suspension group and Adhesion group of HCT116 (n = 5). (B) The comparison of wound healing assay of Suspension group and Adhesion group of HCT116 (n = 10). The red arrows indicate the cells with remarkably high migration capacity.


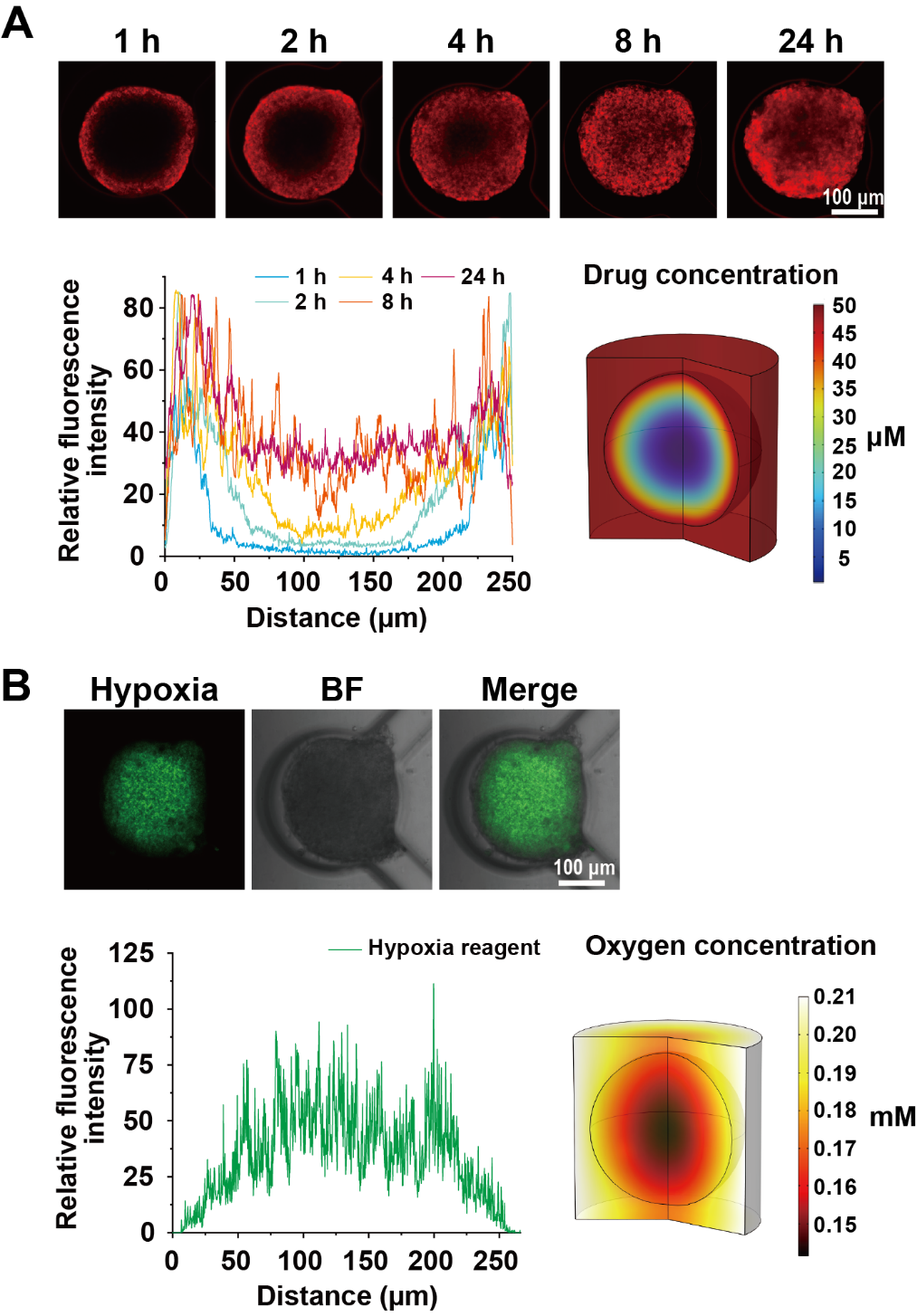


**Figure S14.** (A) Fluorescence images recording the penetration of Rhodamine 6G in the spheroids. The distribution of fluorescence intensity in the radial direction of the sphere. As well as numerical simulation results of 5-FU concentration in spheroids in the chamber when penetration equilibrium is reached. (B) Fluorescence images reflecting the hypoxia within spheroids. The spheroids were labeled with Image-iT Green Hypoxia. The distribution of fluorescence intensity in the radial direction of the sphere. As well as numerical simulation results of oxygen concentration in spheroids in the chamber when penetration equilibrium is reached.


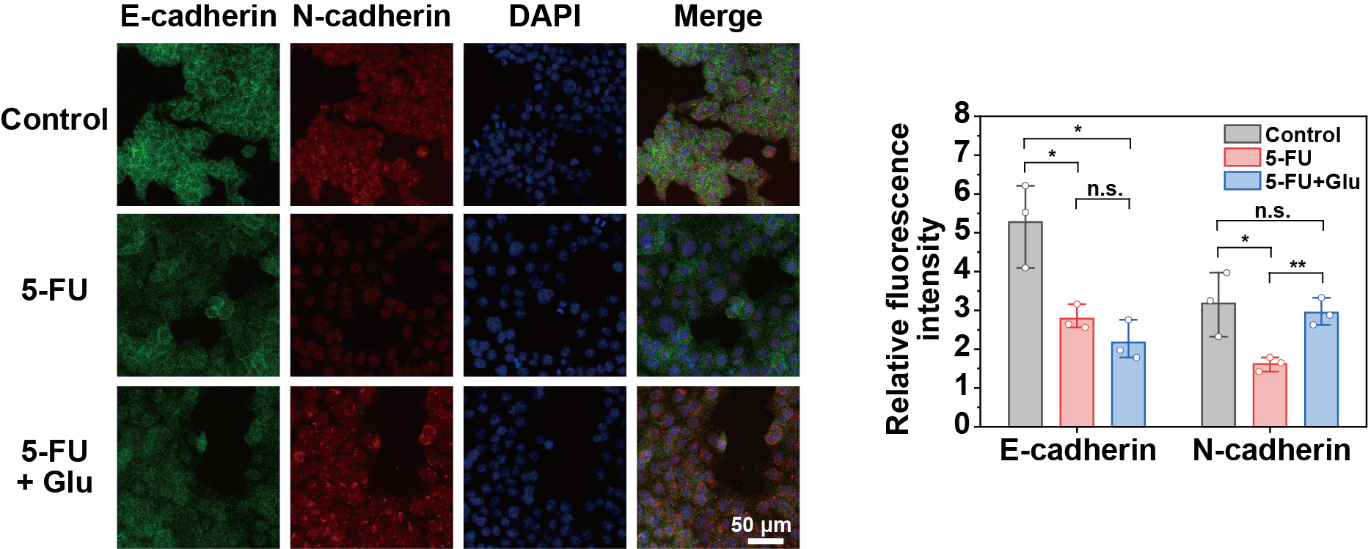


**Figure S15.** Immunofluorescence staining images and statistics showing the expression levels of E-cadherin (green), and N-cadherin (red) in HCT116 cells treated with 5μM 5-FU and 5μM 5-FU with 1mM glutamic acid for 24h as compared to the control group. DAPI (blue) was used to locate the nuclei (n=3).


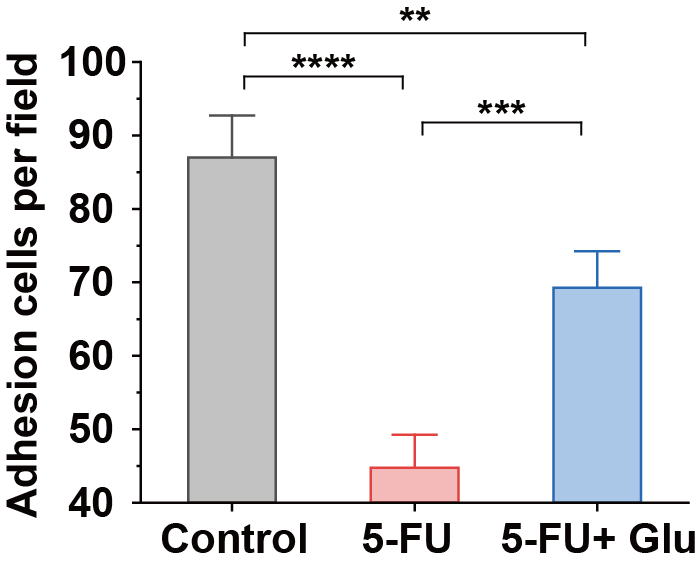


**Figure S16.** Statistical results of the number of adhesion cells in the dynamic cell adhesion assay. Before the experiment, HCT116 cells were treated with 5μM 5-FU and 5μM 5-FU with 1mM glutamic acid for 24h (n = 4). The control group was treated with DMEM complete medium.


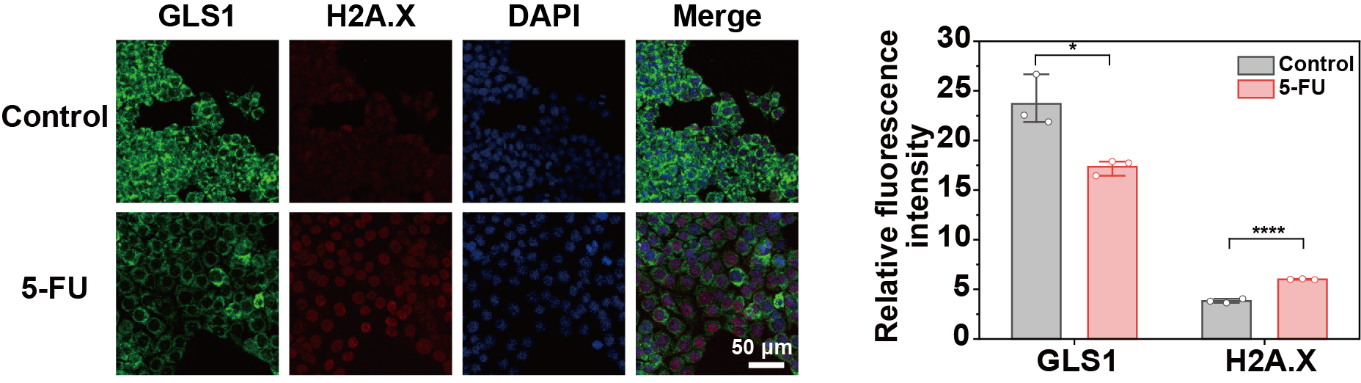


**Figure S17.** Immunofluorescence staining images and statistics showing the expression levels of Glutaminase 1 (green), and Phospho-Histone H2A.X (red) in HCT116 cells treated with 5μM 5-FU for 24h as compared to the control group (n = 3). DAPI (blue) was used to locate the nuclei.


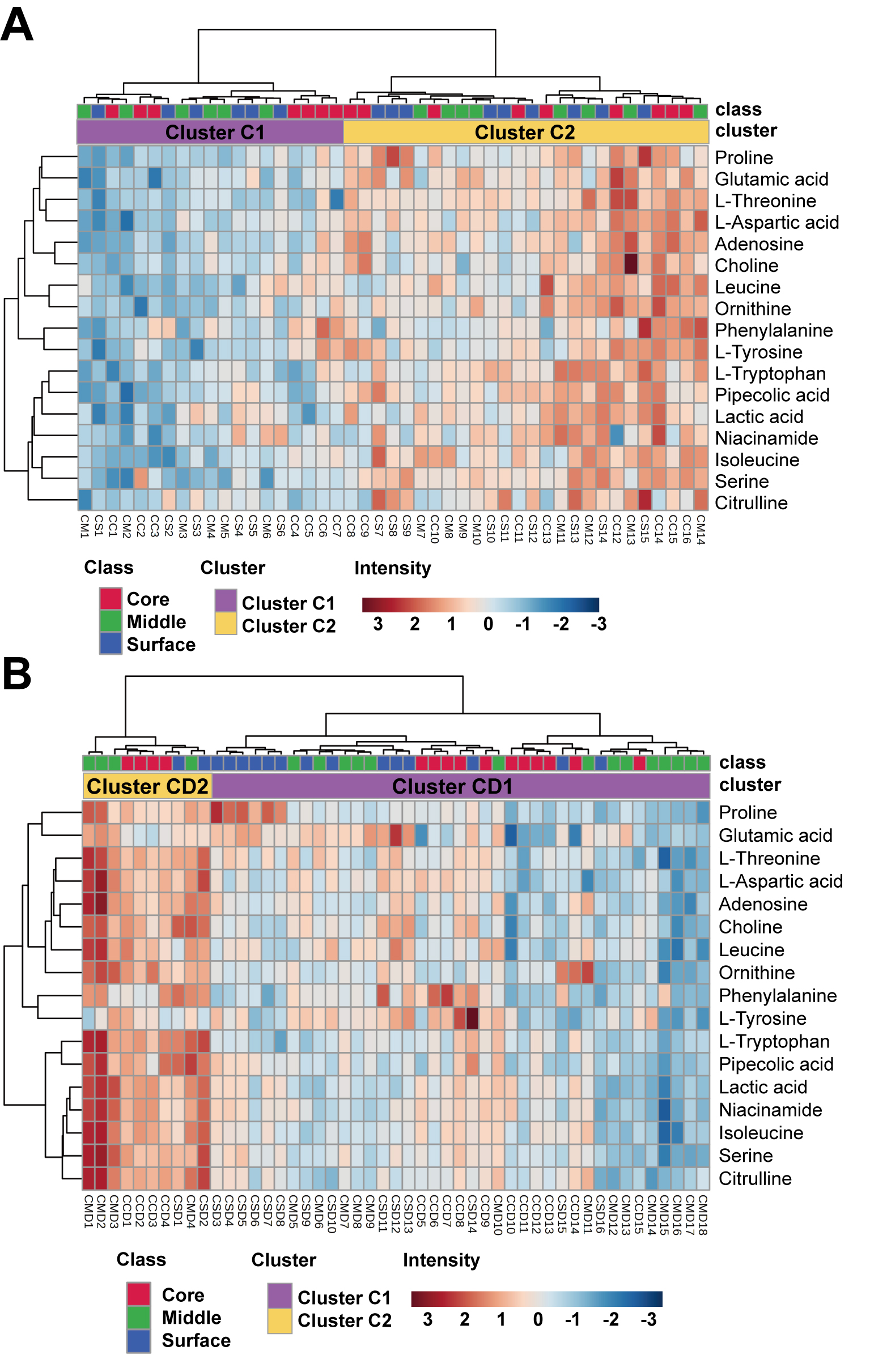


**Figure S18.** Heatmaps of (A) CTC clusters and (B) CTC clusters extracted after drug treatment were both clustered into two clusters. A total of 16 core clusters (CC), 14 middle clusters (CM), 15 surface clusters (CS), 15 core clusters post-drug (CCD), 18 middle clusters post-drug (CMD), and 16 surface clusters post-drug (CSD) were analyzed.


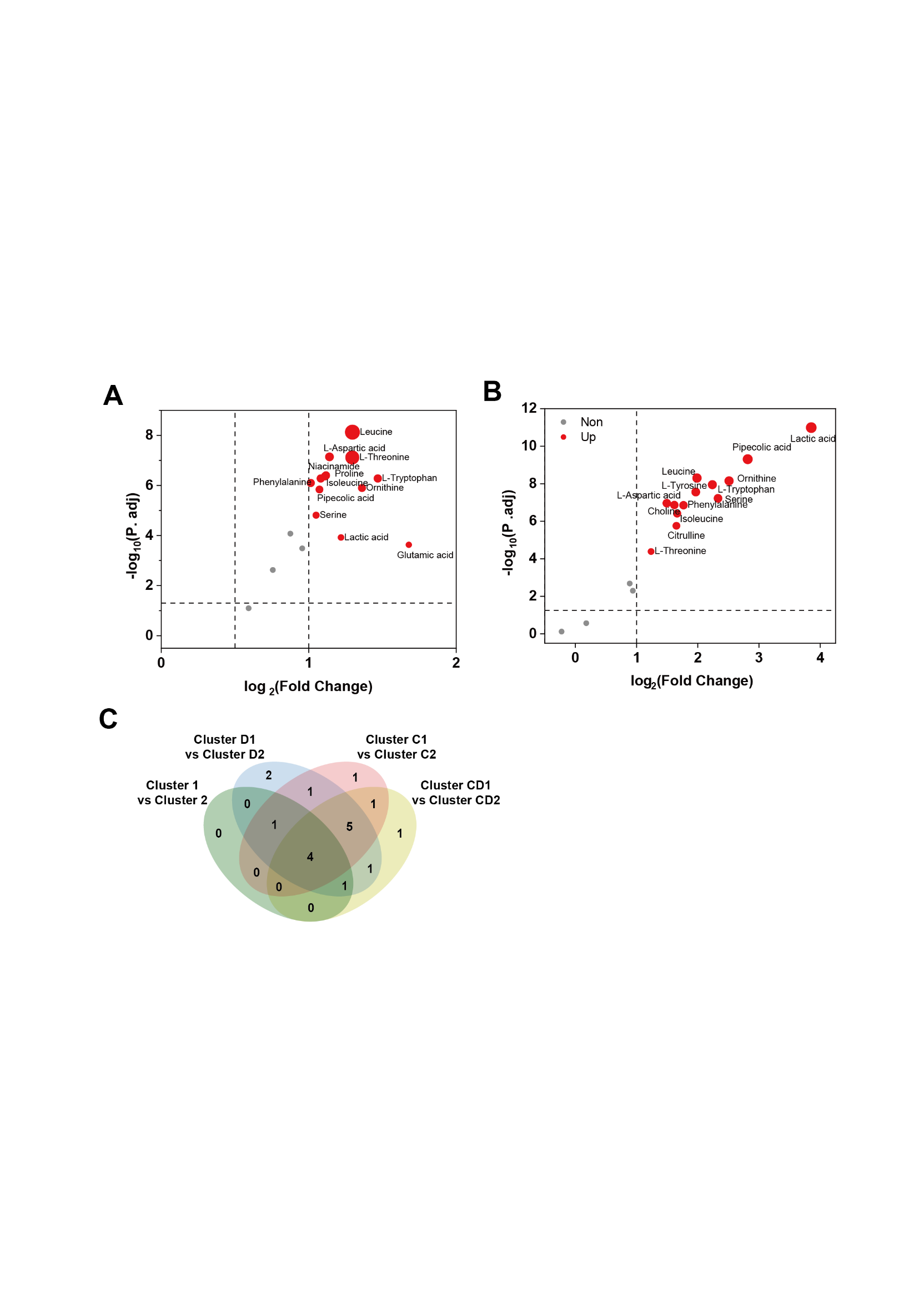


**Figure S19.** Volcano plot showing significantly different metabolites in (A) Cluster C2 compared to Cluster C1 and (B) Cluster CD2 compared to Cluster CD1. (fold change >2, P. adj <0.05). (C) The Venn diagram showing the intersection of the metabolites with significantly differential abundance between Cluster C2 vs. Cluster C1, and Cluster CD2 vs. Cluster CD1.


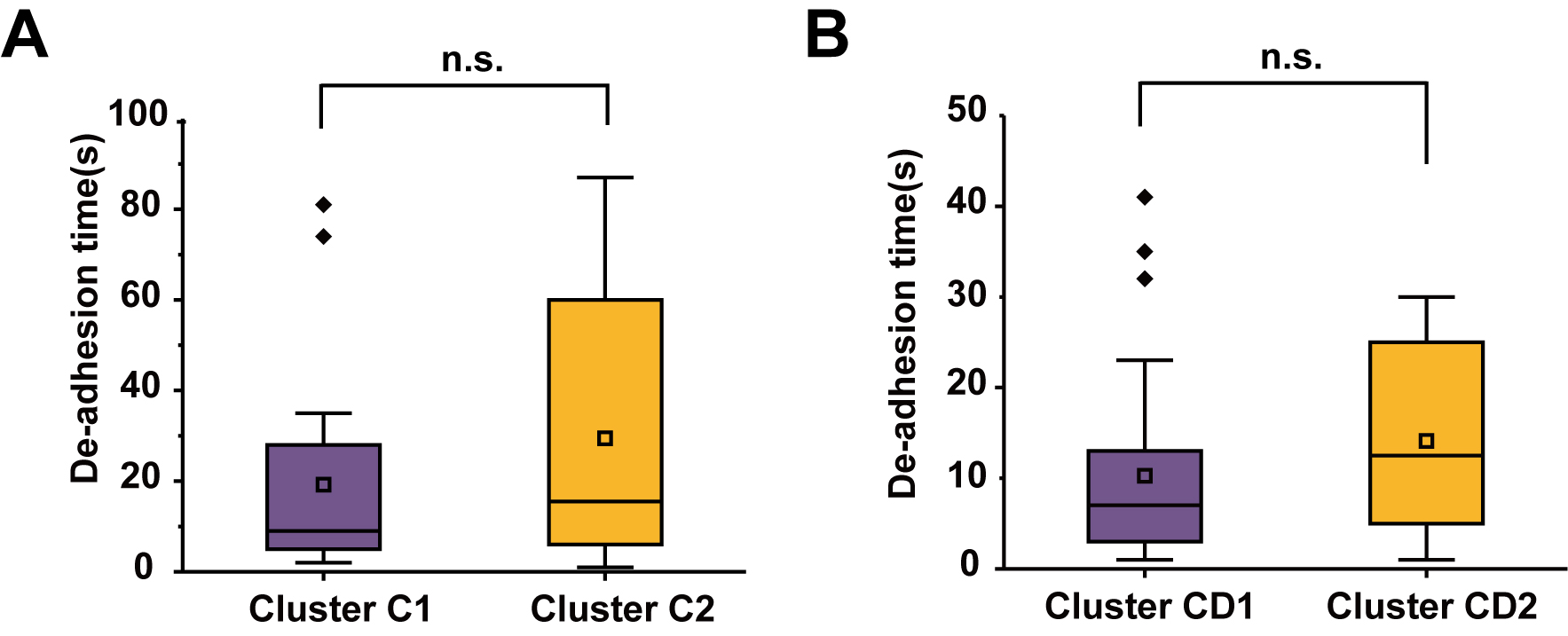


**Figure S20.** Comparison of de-adhesion time between (A) Cluster C1 vs. Cluster C2 (P = 0.2) and (B) Cluster CD1 vs. Cluster CD2 (P = 0.3).


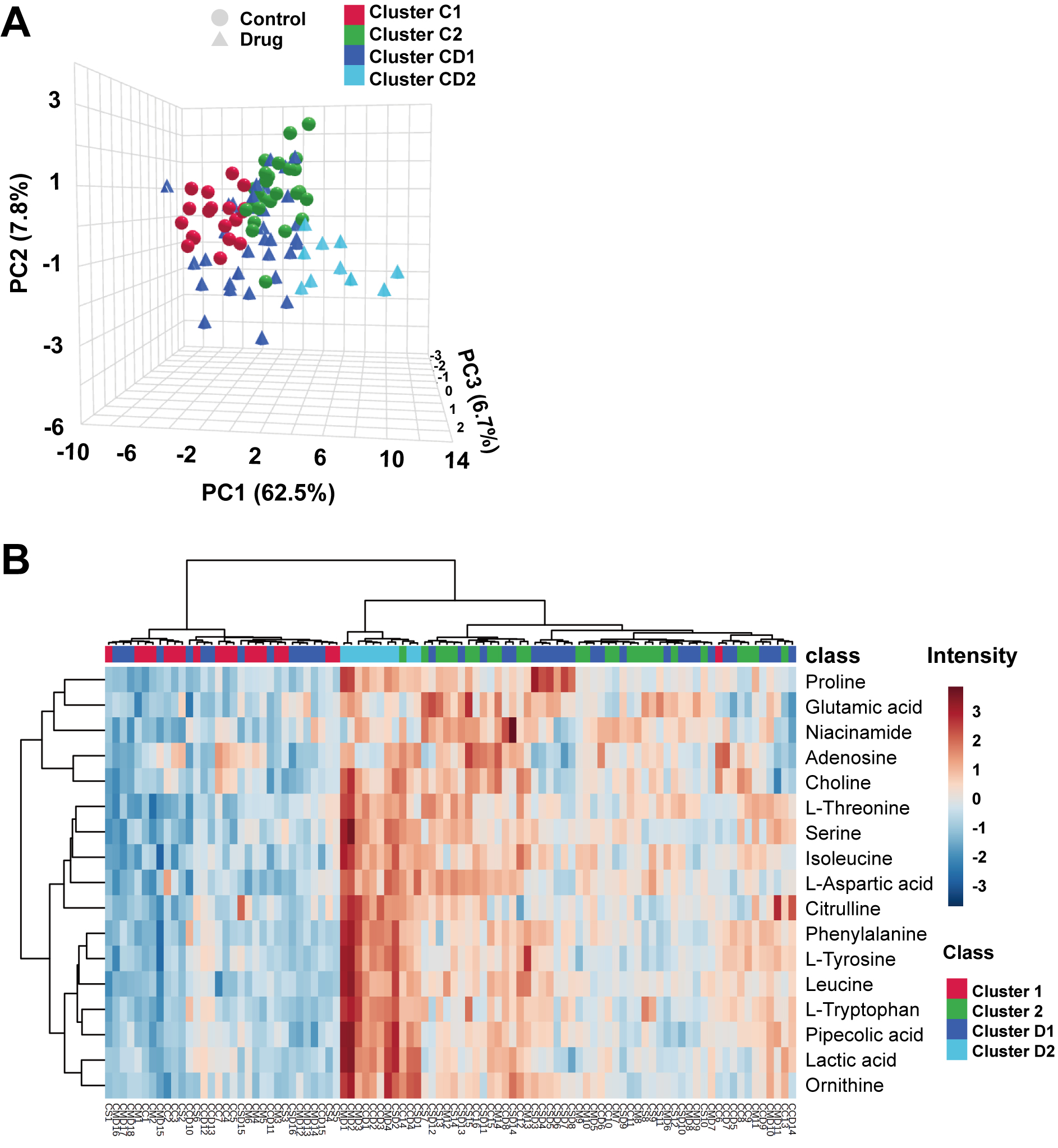


**Figure S21.** (A) 3D interactive PCA plot and (B) heatmap of all CTC cluster samples showing similar metabolic patterns between Cluster C1 and Cluster CD1, as well as Cluster C2 and Cluster CD2.

**Table S1.** The multiple reaction monitoring (MRM) mode parameters of 17 target metabolites.

| **No.** | **Chemical name**  **(positive/negative mode)** | **MRM**  **(m/z)** | **CE**  **(*eV*)** |
| --- | --- | --- | --- |
| 1 | L-Aspartic acid (+) | 134.00>74.05 | -15 |
| 2 | Serine (+) | 105.90>60.10 | -12 |
| 3 | L-Threonine (+) | 120.10>74.15 | -13 |
| 4 | Glutamic acid (+) | 147.90>84.10 | -17 |
| 5 | Citrulline (+) | 176.10>70.05 | -25 |
| 6 | Ornithine (+) | 133.10>70.10 | -18 |
| 7 | Proline (+) | 116.10>70.15 | -18 |
| 8 | Choline (+) | 104.10>60.05 | -22 |
| 9 | Pipecolic acid (+) | 130.10>84.05 | -18 |
| 10 | Niacinamide (+) | 123.10>80.05 | -23 |
| 11 | L-Tyrosine (+) | 182.10>136.10 | -15 |
| 12 | Adenosine (+) | 268.10>136.05 | -18 |
| 13 | Isoleucine (+) | 132.10>69.15 | -19 |
| 14 | Leucine (+) | 132.10>30.05 | -18 |
| 15 | Phenylalanine (+) | 166.10>103.10 | -29 |
| 16 | L-Tryptophan (+) | 205.10>188.15 | -12 |
| 17 | Lactic acid (-) | 89.30>89.05 | 7 |

**Table S2.** Parameters used in the simulation of 5-fluorouracil (5-FU) and oxygen concentration distributions.

| **Parameter** | **Value (5-FU)** | **Value (Oxygen)** |
| --- | --- | --- |
| $D_{w},$ diffusion coefficient in water | 1.023 × 10^-9^ m^2^/s^[2]^ | 2.8 × 10^-9^ m^2^/s^[1]^ |
| $D_{c},$diffusion coefficient in tumor spheroid | 3.57 × 10^-13^ m^2^/s^[3]^ | 2.5 × 10^-9^ m^2^/s^[1]^ |
| $V_{max},$ maximum uptake rate of cells | 4.68 × 10^-21^ mol/cell/s^[4]^ | 4.1 × 10^-17^ mol/cell/s^[1]^ |
| $K_{m},$Michaelis–Menten constant | 8.04 × 10^-4^ mol/m^3 [4]^ | 1.294 × 10^-2^ mol/m^3[1]^ |
| $N_{\text{cell, }}$cell density in tumor spheroids | 1.1× 10^-9^ cells/mL | 1.1× 10^-9^ cells/mL |
| $C_{0},$concentration at the boundary surface | 50 μM | 0.21 mM |

**Movie S1.**

Example of stable rolling mode arrest behavior of individual CTC under fluid shear stress. The flow rate was set at 20 μL/min.

**Movie S2.**

Example of rolling-stopping-detachment mode arrest behavior of individual CTC under fluid shear stress. The flow rate was set at 20 μL/min.

**Movie S3.**

Example of rolling-stopping-detachment mode arrest behavior of CTC cluster under fluid shear stress. The flow rate was set at 20 μL/min.

**Movie S4.**

Example of cluster segregation mode arrest behavior of CTC cluster under fluid shear stress. The flow rate was set at 20 μL/min.

**Movie S5.**

Example of stable adhesion mode arrest behavior of individual CTC under fluid shear stress. The flow rate was set at 10 μL/min.

References

[1] H.-G. Yi, Y. H. Jeong, Y. Kim, Y.-J. Choi, H. E. Moon, S. H. Park, K. S. Kang, M. Bae, J. Jang, H. Youn, S. H. Paek, D.-W. Cho, *Nat. Biomed. Eng.* **2019**, *3*, 509.

[2] L. M. P. Verissimo, I. Cabral, A. M. T. D. P. V. Cabral, G. Utzeri, F. J. B. Veiga, A. J. M. Valente, A. C. F. Ribeiro, *J. Chem. Thermodyn.* **2021**, *161*, 106533.

[3] M. Boodaghi, S. Libring, L. Solorio, A. M. Ardekani, *J. Controlled Release* **2021**, *340*, 60.

[4] K. Ueda, T. Nakamura, S. Tanaka, M. Hosokawa, S. Iwakawa, K. Ogawara, *Drug Metab. Pharmacokinet.* **2020**, *35*, 124.
